# Wide-Field Fundus Autofluorescence Patterns in a Polish Cohort of Genotyped Patients with Phenotype of Retinitis Pigmentosa, Usher Syndrome and Cone–Rod Dystrophy

**DOI:** 10.3390/ijms27219744

**Published:** 2026-10-30

**Authors:** Agata Pietras-Baczewska, Beata Gajda-Deryło, Katarzyna Ognik, Robert Rejdak, Katarzyna Nowomiejska

**Affiliations:** 1 Department of General and Pediatric Ophthalmology, Medical University of Lublin, 20-079 Lublin, Poland; agatapie@gmail.com (A.P.-B.); beata.gajdaa@gmail.com (B.G.-D.); robert.rejdak@umlub.edu.pl (R.R.); 2 Department of Biochemistry and Toxicology, Faculty of Animal Sciences and Bioeconomy, University of Life Sciences in Lublin, 20-950 Lublin, Poland; katarzyna.ognik@up.edu.pl

**Keywords:** fundus AF, inherited retinal dystrophies, phenotype

## Abstract

To investigate different wide-field fundus autofluorescence (FAF) patterns in a large cohort of Polish patients with inherited retinal dystrophies (IRDs) of different genotypes sorted into groups of at least 5 patients. A total of 103 patients with IRDs and phenotype of retinitis pigmentosa, Usher syndrome and cone–rod dystrophy were included and divided into groups of at least 5 patients based on the outcome of genetic analysis. Ultra-wide field FAF imaging was reviewed to determine patterns of FAF. Ten groups of the same genotype and three patterns of FAF have been distinguished. In the most prevalent *USH2A* group (26 cases), macular hyperautofluorescence (AF) ring combined with mottled hypo-AF in the periphery was present in 19 cases, the hyper-AF ring alone (including one double ring) was present in 5 cases, while the peripapillary sparing appeared in 2 cases. In the *ABCA4* group (22 cases), mottled hypo-AF pattern in the posterior pole and mid-periphery was most prevalent (13 cases), additional peripapillary sparing was present in 4 cases, central hyper-AF ring was present in 3 cases, and characteristic flecks were present in 2 cases. In the *RPGR* group (15 patients), the most prevalent pattern was a combination of hyper-AF ring and mottled hypo-AF in the periphery (13 cases), while in 2 cases only hypo-AF mottling was present. In *PROM1* gene variants (7 patients), mottled hypo-AF in the posterior pole was again the most dominant pattern (6 patients), and there was one case of combined hyper-AF ring and hypo-AF in the periphery. All *NR2E3* (5 patients), *RP1* (5 patients), *RHO* (7 patients), *CDHR1* (5 patients), *PRPF31* (5 patients) and *EYS* (6 patients) gene pathogenic variants also manifested with hyper-AF ring in the center and patchy hypo-AF spots in the periphery. Only one of the *NR2E3* patients demonstrated a double ring. The most prevalent pattern across all groups, excluding *ABCA4* and *PROM1* groups, is central hyper-AF ring together with patchy hypo-AF in the periphery (72 cases—70%). *ABCA4* and *PROM1*-related RP groups revealed mostly hypo-AF mottled patterns in the posterior pole and periphery, without hyper-AF ring. FAF patterns are very heterogenous, but there are some distinctive features in different genotypes of IRDs.

## 1. Introduction

Fundus autofluorescence (FAF) imaging is a non-invasive retinal imaging technique that creates a topographic map of emission from naturally occurring fluorophores in the ocular fundus following excitation at a defined wavelength, providing an indirect readout of RPE/photoreceptor complex metabolic and structural status. It is used clinically to diagnose, phenotype, and monitor a wide range of retinal disorders, particularly those involving the outer retina and retinal pigment epithelium (RPE) [1,2]. FAF is a particularly useful diagnostic tool in inherited retinal dystrophies (IRDs) as it provides a rapid, non-invasive “metabolic map” of RPE/photoreceptor complex dysfunction (mainly lipofuscin signal on short-wavelength FAF; melanin-related signal on near-infrared FAF). Changes detected by FAF imaging often precede or are better delineated than those seen on color fundoscopy, and FAF imaging can be used to monitor disease progression and treatment response [3]. Ultra-widefield coverage is useful because pathology associated with IRDsoften extends beyond the posterior pole.

Lipofuscin accumulates over time in the lysosomal storage bodies of RPE cells and serves as the primary fluorophore in FAF. It is produced as part of the visual cycle in the outer segments of photoreceptors and is composed of a mixture of bisretinoids, including A2E, all-trans-retinal dimer, and related isomers [4,5]. The emission spectra of these compounds, which peak at roughly 600 nm, formed the basis for FAF imaging [6]. Pathologic disruption of the visual cycle in photoreceptors, as well as the resulting reduction in bisretinoid production or excessive accumulation of bisretinoids in the RPE, are signs of disease detectable on FAF [7].

Optos ultra-widefield systems are confocal scanning laser ophthalmoscopes (cSLOs) that can acquire ~200° retinal images using an ellipsoidal mirror optical design. Optos ultra-wide field (UWF-FAF) imaging uses an excitation wavelength of approximately 532 nm (green) and an emission detection range of approximately 570–780 nm. Optos may have a 488 nm laser, but in common configurations it is used for fluorescein [8]. FAF images are usually interpreted in terms of relative hypoautofluorescence (hypo-AF) and hyperautofluorescence (hyper-AF). Hypo-AF commonly correlates with RPE loss or atrophy, signal blockage due to hemorrhage, pigment, or exudate, or the absence of fluorophores. Hyper-AF is often interpreted as an increased fluorophore load (e.g., lipofuscin), stressed and altered RPE, or photoreceptors at transition zones [4,6].

IRDs are a heterogeneous group of genetically mediated disorders in which dysfunction and/or degeneration of photoreceptors (rods and/or cones), retinal pigment epithelium (RPE), and sometimes inner retina or choroid leads to progressive (or occasionally stationary) visual impairment. Clinically, they are defined by phenotype, inheritance pattern, objective functional and structural testing, and increasingly by a molecular diagnosis. IRDs can show autosomal-dominant, autosomal-recessive, X-linked, or mitochondrial inheritance. Sporadic cases, including those associated with de novo mutations, also occur. IRDs can be nonsyndromic, with manifestations limited to the eyes, or syndromic, with systemic involvement such as hearing loss, renal disease, obesity, or features of ciliopathy or neuropathy. Over 300 different genes have been implicated in the pathogenesis of IRDs, posing a significant challenge in diagnosis, monitoring of disease, and identification of biomarkers for clinical trial outcomes [9].

Instead of the fact that Poland has a population of about 37 million, IRDs are not well-characterized in this country and only a few papers describe the genotypes of patients in small cohorts with Stargardt disease, RP, and various IRDs [10,11], RP [12,13] and different IRDs [14]. The clinical characteristics of Polish patients with IRDs have not been extensively characterized to date.

The aim of this study was to describe the FAF patterns in a group of 103 genotyped Polish patients with phenotype of retinitis pigmentosa (RP), Usher syndrome and cone–rod dystrophy.

## 2. Results

Detailed description of the FAF classification and a table of pathogenic variants are provided in the Supplementary Material.

### 2.1. USH2A Gene

Pathogenic variants in the *USH2A* gene were found in 26 patients in the cohort. Consequently, this was the largest and most homogeneous group. Among patients with identified USH2A variants, missense variants dominated the pathogenic variant spectrum (~60%). c.11864 G>A is the major recurrent *USH2A* allele—6 patients (≈23% of patients). A deletion of exons 21–23 was the most important recurrent CNV, identified in 5 participants. Biallelic *USH2A* mutations are present in 13 cases, providing compelling evidence for autosomal-recessive USH2A-associated disease. No single compound-heterozygous genotype recurs, indicating substantial allelic heterogeneity despite a few recurrent founder alleles.

**Figure 1:**
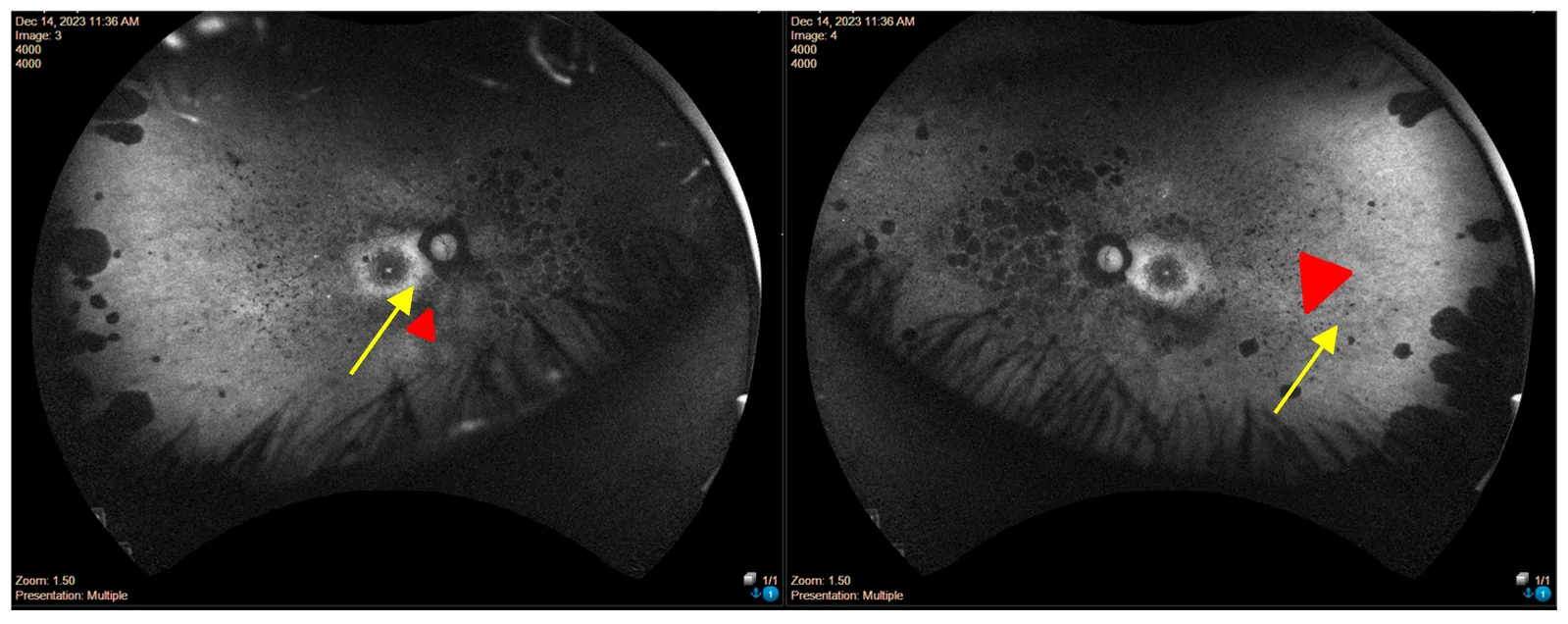
Fundus autofluorescence image of both eyes of patient LUC 29 with *USH2A*-linked retinitis pigmentosa. Hypo-AF fovea (red arrowhead) surrounded by hyper-AF complete ring (yellow arrow). Retinal periphery presented punctate hypoautofluorescent spots distributed predominantly throughout the midperipheral quadrant.

**Figure 2:**
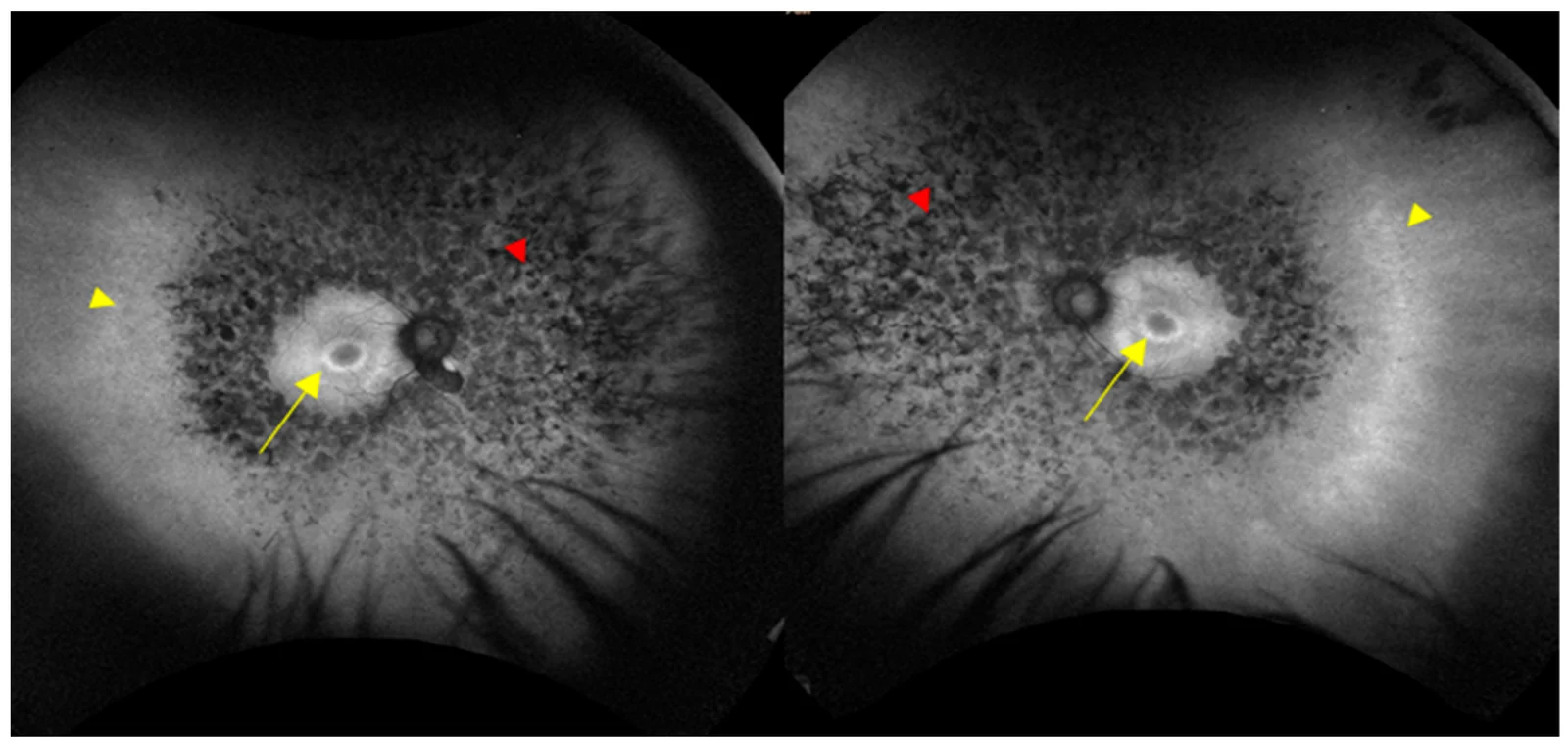
Fundus autofluorescence images of both eyes of patient LUC 153 with *USH2A*-linked retinitis pigmentosa likely due to pathogenic deletion of coding exons 21–23. Patient A hyper-AF parafoveal (yellow arrow) and pericentral hyperautofluorescent ring (yellow arrowhead) “double ring”, diffuse homogeneous hypoautofluorescence (red arrowhead), and preserved distal periphery.

In *USH2A* patients with RP or Usher syndrome phenotype, FAF showed a single, complete macular hyperautofluorescent ring pattern alone (5 cases) or in combination with a hypo-AF (patch) (19 cases) (Figure 1). A double hyper-AF ring was present in one case (Figure 2). The retinal periphery presented punctate hypo-AF spots distributed predominantly throughout the midperipheral and peripapillary areas. Peripapillary sparing was present in 2 cases.

### 2.2. ABCA4 Gene

**Figure 3:**
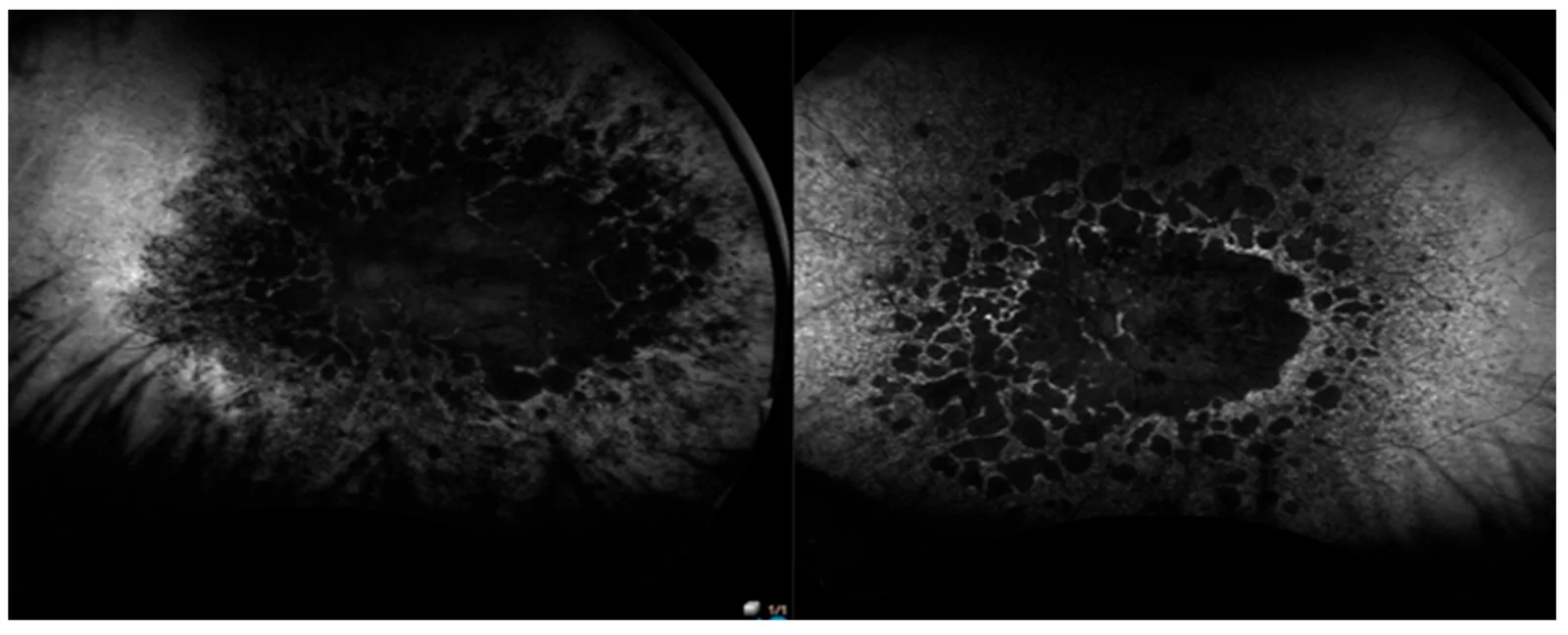
Fundus autofluorescence image of patient LUC 92 with *ABCA4*-linked retinitis pigmentosa due to pathogenic variants: c.5512 C>T, c.3113 C>T and c.1622 T>C. Macular and midperipheral retina presented focal mottled hypo-AF.

Out of the 22 patients belonging to the *ABCA4* gene group, 19 patients exhibited the RP phenotype and 3 had CRD. The *ABCA4* variant spectrum was enriched for missense variants. No pathogenic CNVs or clearly truncating pathogenic variants (frameshift or nonsense) were observed in this dataset. Several recurrent splicing defects contributed to the disease burden. The *ABCA4* spectrum is dominated by a small number of recurrent alleles—c.1622 T>C (12 patients), c.3113 C>T (11 patients), c.454 C>T (4 patients)—whereas other alleles were observed twice. The genotype c.1622 T>C/c.3113 C>T was the predominant disease-associated combination, occurring in multiple unrelated patients and suggesting a possible founder effect or enrichment of recurrent alleles in the studied population. Most patients had biallelic pathogenic/likely pathogenic *ABCA4* variants, consistent with the expected autosomal-recessive inheritance of *ABCA4*-associated retinopathy. A small subset of patients carried only a single pathogenic *ABCA4* allele, indicating that additional pathogenic variants (e.g., deep-intronic variants, structural variants, or undetected second alleles) may remain to be identified (Supplementary Material). In *ABCA4*-associated retinopathy, the macular and midperipheral retina showed a focal hypo-AF pattern in 14 of the 22 patients, making this the largest subgroup. A representative example is shown in Figure 3.

**Figure 4:**
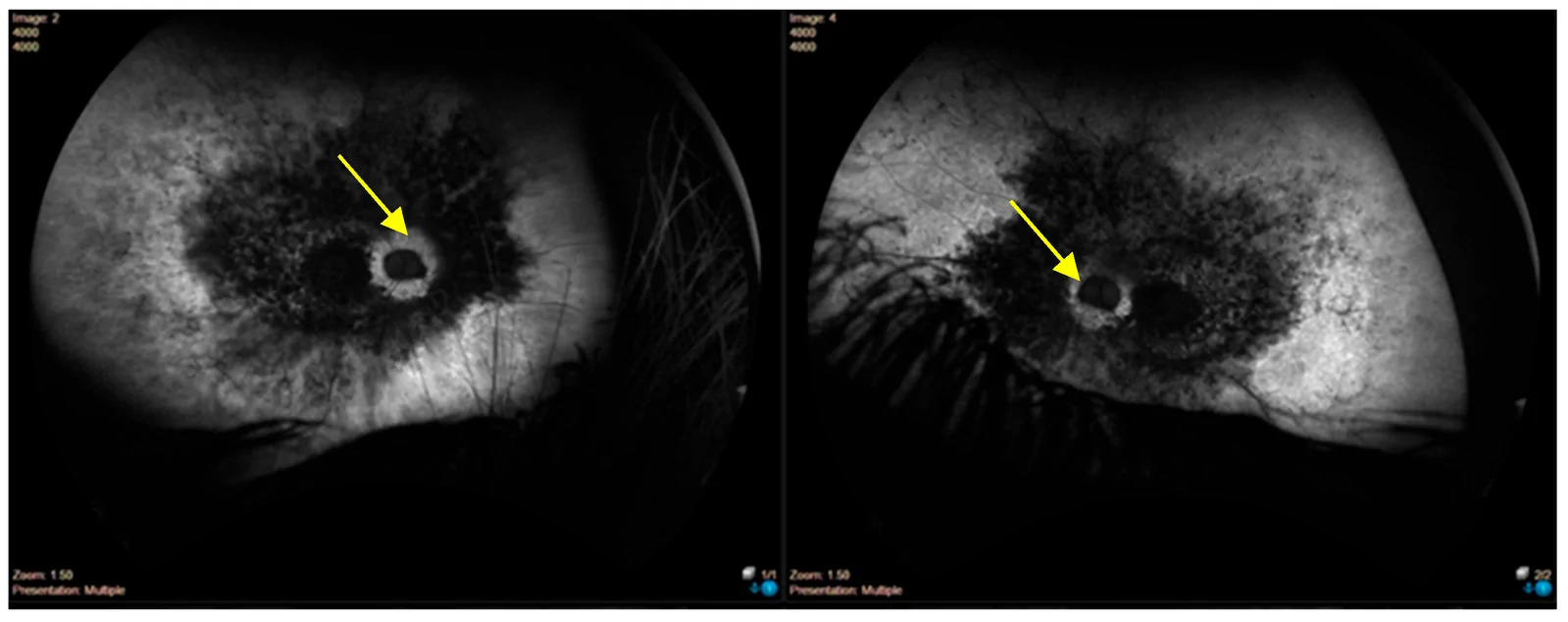
Fundus autofluorescence image of both eyes in patient LUC134 with *ABCA4*-linked retinitis pigmentosa pathogenic variants: c.319 C>T, c.1622 T>C and c.3113 C>T. Preserved fluorescence around optic disk—peripapillary sparing (yellow arrow).

Peripapillary sparing, defined as relative preservation of the AF signal around the optic disk, was additionally observed in four patients in this group (Figure 4).

**Figure 5:**
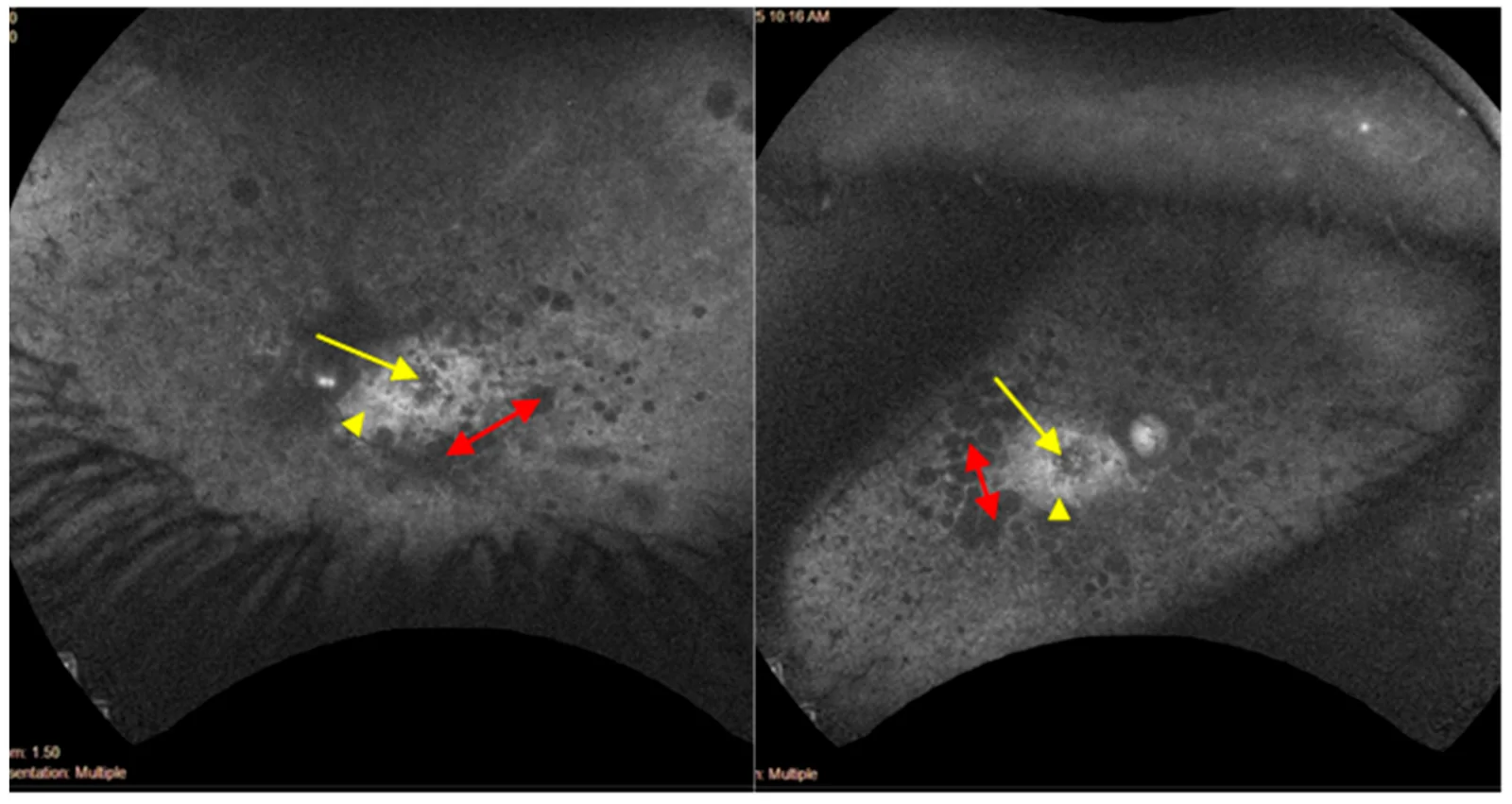
Fundus autofluorescence images of patient LUC 34 with *ABCA4*-linked retinitis pigmentosa due to c.4253+43 G>A pathogenic variant. Hyper-AF ring in the macula (yellow arrows), pericentral hyperautofluorescent ring (yellow arrowhead). Midperipheral patchy hypo-AF (red double-ended arrow).

Macular involvement was additionally observed in three patients, with central hypo-AF surrounded by a hyper-AF border (hyper-AF ring) or the appearance of atrophy with a junctional zone (Figure 5).

**Figure 6:**
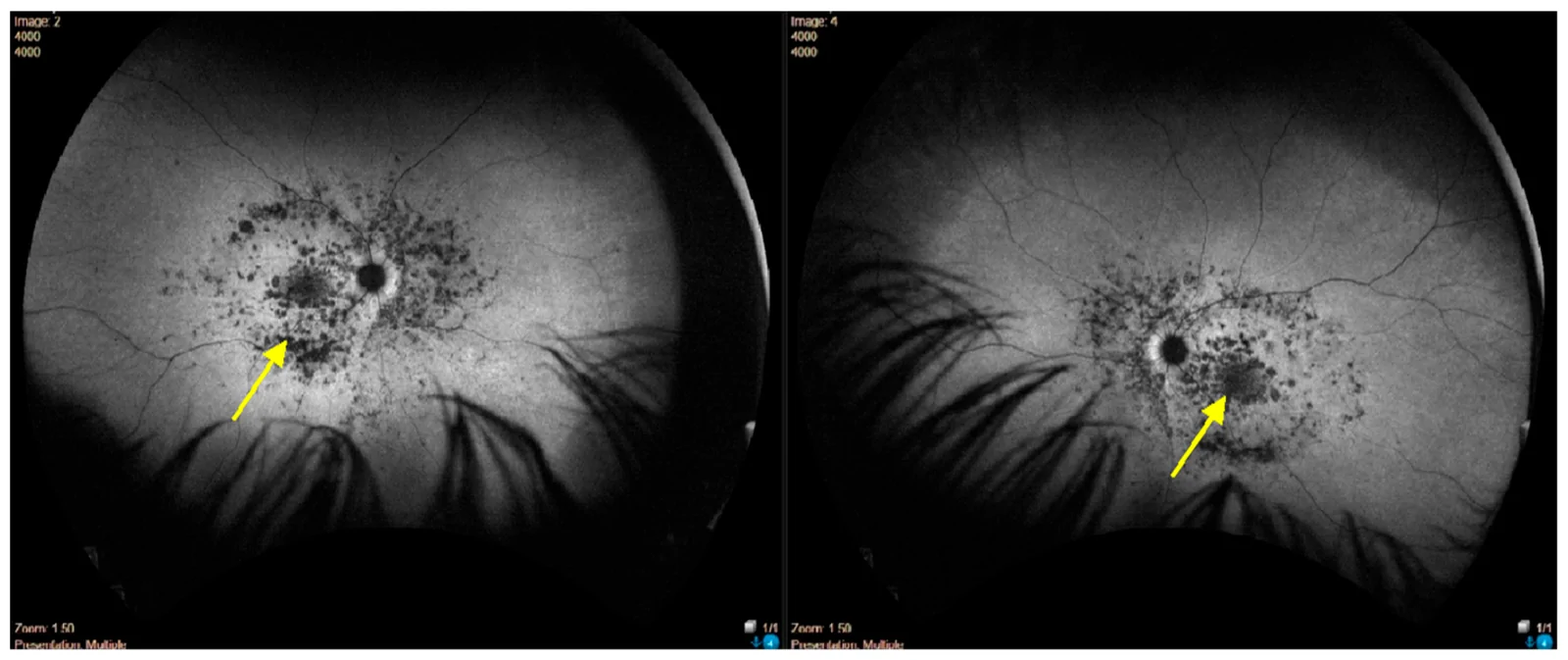
Fundus autofluorescence images of both eyes in patient LUC 86 with *ABCA4*-linked retinitis pigmentosa—freckles of hypo-AF in perimacular area (yellow arrow)—concentrated along the vascular arcades, with preserved fluorescence in the distal periphery.

Two patients exhibited what are known as classic Stargardt-like flecks, which are speckled hyper-AF or hypo-AF in the macular and perimacular regions (Figure 6).

### 2.3. RPGR Gene

**Figure 7:**
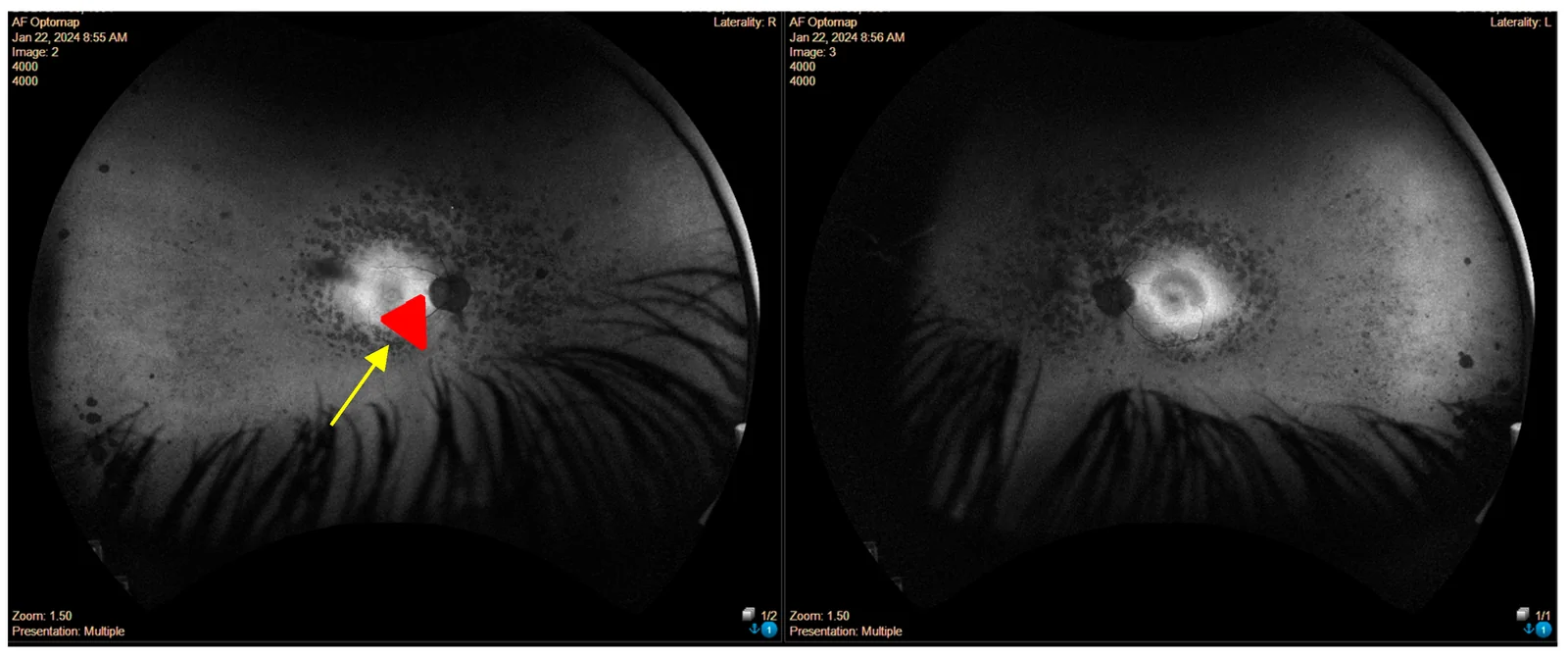
Fundus autofluorescence images of eyes in patients LUC75 with *RPGR*-linked retinitis pigmentosa. Hyperautofluorescent ring (yellow arrow) surrounding hypofluorescent macula (red arrowhead). Patchy speckled hypoautofluorescence in the midperiphery.

The *RPGR* group consists of 15 patients carrying *RPGR*-related pathogenic or likely pathogenic variants. The cohort demonstrated marked RPGR allelic heterogeneity, with only c.194G>A observed in two patients, while all other variants were identified in one patient each. The mutational spectrum was dominated by frameshift and splice-site variants, consistent with loss-of-function mechanisms affecting *RPGR* function. In 13 patients with RPGR variants, FAF showed central confluent or patchy macular atrophy, appearing as hypo-AF surrounded by a hyper-AF ring, together with peripheral hypo-AF (Figure 7). The remaining two patients showed mottled peripheral hypo-AF.

### 2.4. PROM1 Gene

**Figure 8:**
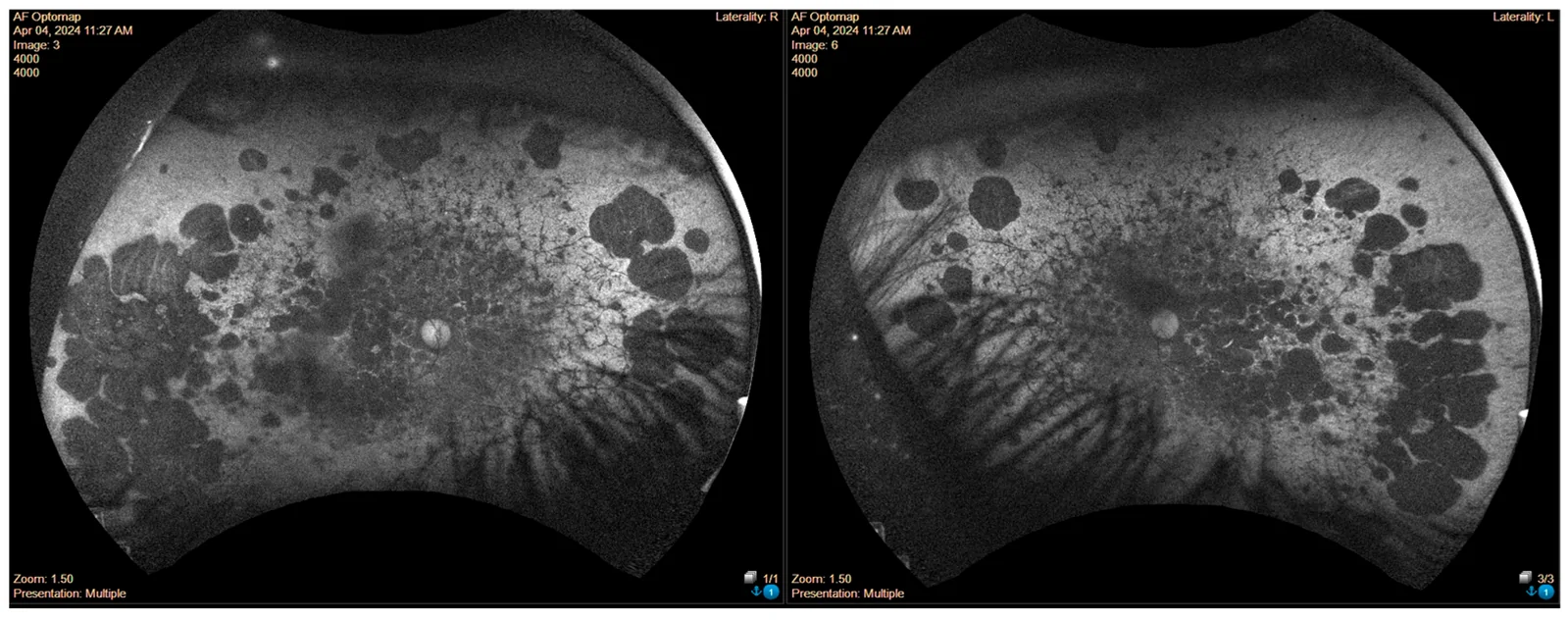
Fundus autofluorescence images of eyes in patient LUC114 with *PROM1*-linked retinitis pigmentosa. Round, sharply demarcated hypoautofluorescent spots in the peripheral retina.

Seven patients carried *PROM1* variants, with c.1301+2 T>C (four of seven patients, 57%) and c.1902 C>G (three of seven patients, 42%) representing the most recurrent disease-associated alleles. The variant spectrum consisted predominantly of missense changes together with one recurrent splice-site variant. In all patients with PROM1 variants, the predominant FAF pattern was mottled hypo-AF in the midperiphery and periphery (Figure 8). In one patient, this was accompanied by a central hyper-AF ring.

### 2.5. NR2E3 Gene

**Figure 9:**
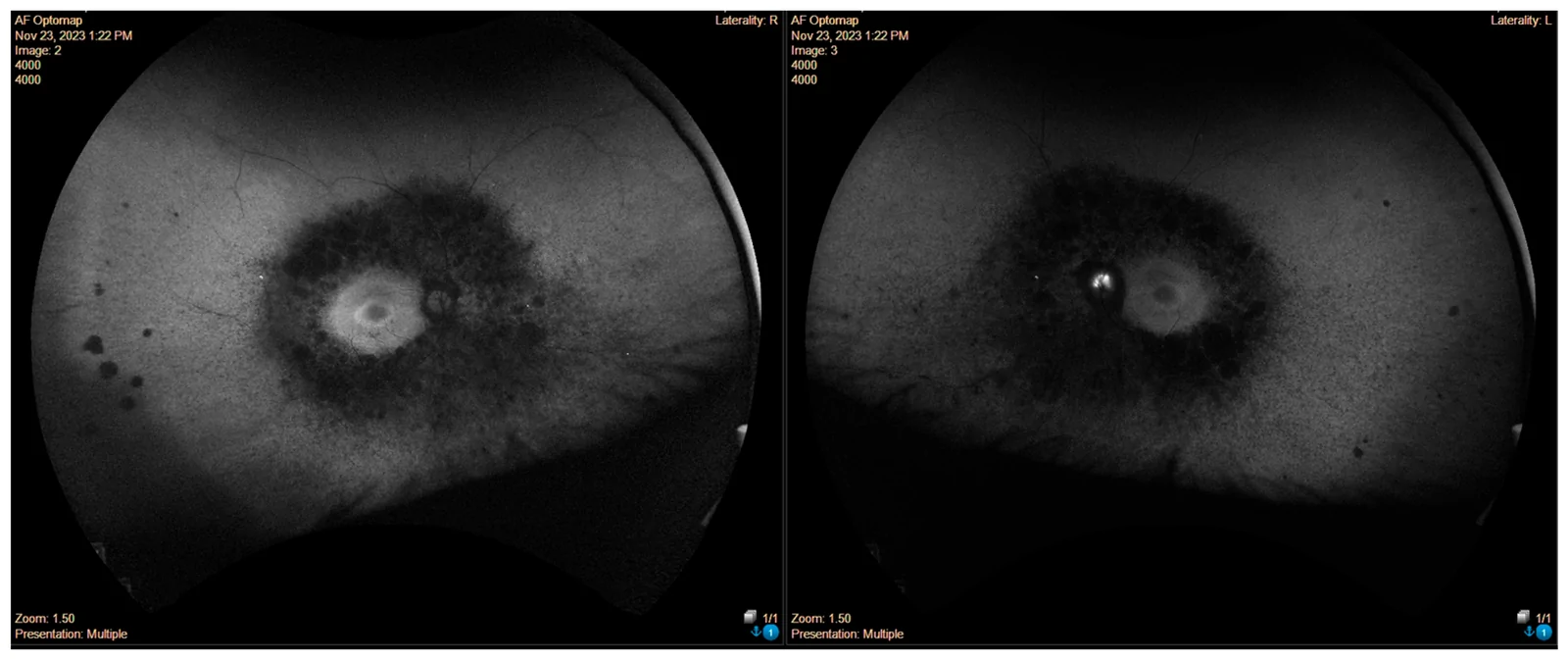
Fundus autofluorescence images of both eyes in patient LUC4 with *NR2E3*-linked retinitis pigmentosa. Present pathogenic c.481delA and c.227 G>A. There is a perimacular double hyper-AF ring and diffuse hypo-AF in the midperiphery.

Five patients carried *NR2E3*-related pathogenic variants. The NR2E3 variant c.481delA was the predominant disease-associated allele, identified in 4 patients. The pathogenic spectrum was dominated by frameshift variants, indicating a major contribution of loss-of-function alleles in this cohort. Two patients had biallelic pathogenic *NR2E3* variants consistent with autosomal-recessive disease, whereas three patients carried only a single pathogenic allele. The high recurrence of c.481delA suggests that this variant may represent a recurrent or founder allele within the studied population. *NR2E3*-RP eyes showed a parafoveal double hyper-AF ring like other RP genotypes. The central retina was relatively preserved, whereas the perifoveal region and midperiphery were more affected and showed patchy hypo-AF. The inner hyper-AF ring was perimacular, and the outer ring was located along the vascular arcades. An inner perimacular hyper-AF ring, and an outer ring were located along the vascular arcades. There was one case of double hyper-AF ring (Figure 9).

### 2.6. RP1 Gene

**Figure 10:**
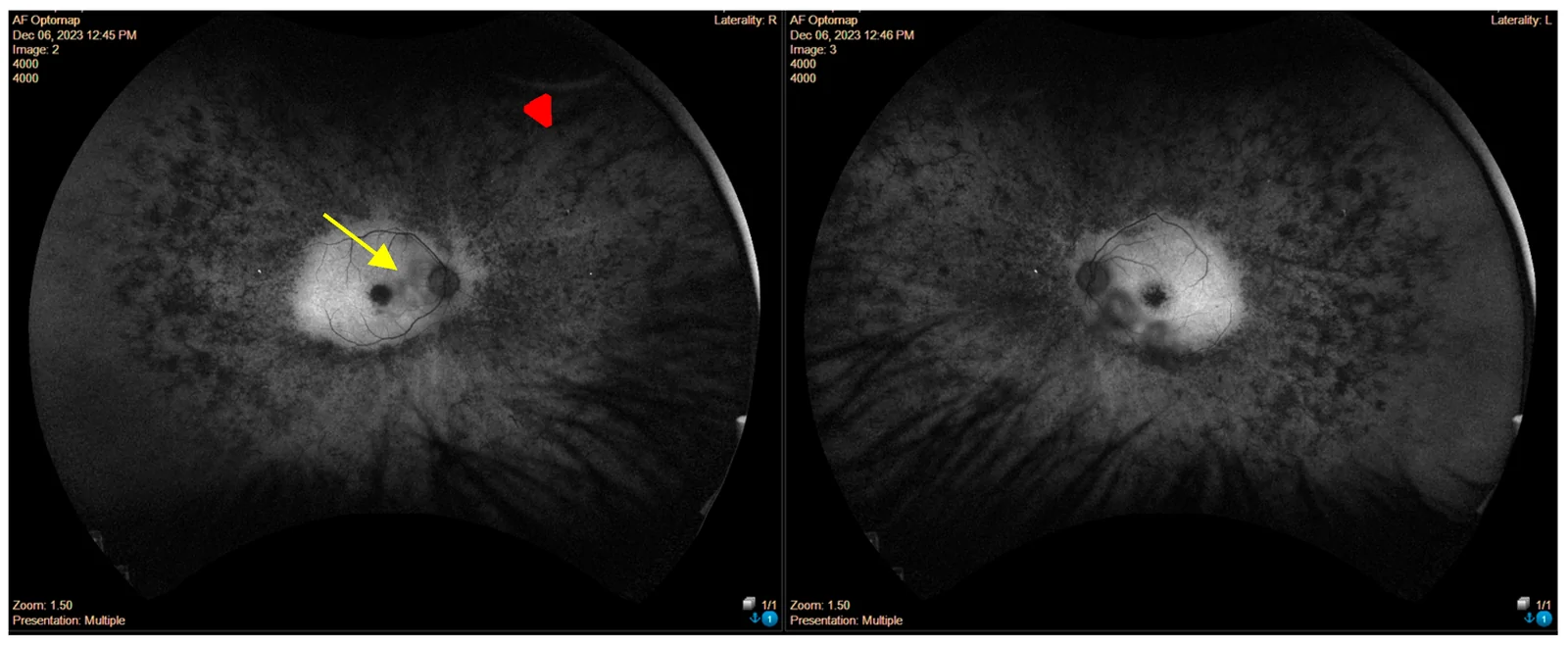
Fundus autofluorescence images of both eyes in patient LUC 173 with *RP1*-linked retinitis pigmentosa. Hyperautofluorescent perimacular area (yellow arrow), central hypoautofluorescent macula, and round, sharply demarcated hypoautofluorescent spots in the peripheral retina (red arrowheads).

Five patients carried *RP1*-related variants. The RP1 variants c.2029C>T and c.2173_2174delinsG were each identified in two unrelated individuals. The cohort demonstrated a heterogeneous mutational spectrum including missense, truncating, and splice-site variants. Three patients carried additional variants in genes associated with other retinal diseases. In *RP1*-associated RP, FAF showed macular atrophy surrounded by a complete perimacular hyper-AF ring. Peripheral hypo-AF ranged from mottled changes to confluent geographic areas (Figure 10).

### 2.7. RHO Gene

**Figure 11:**
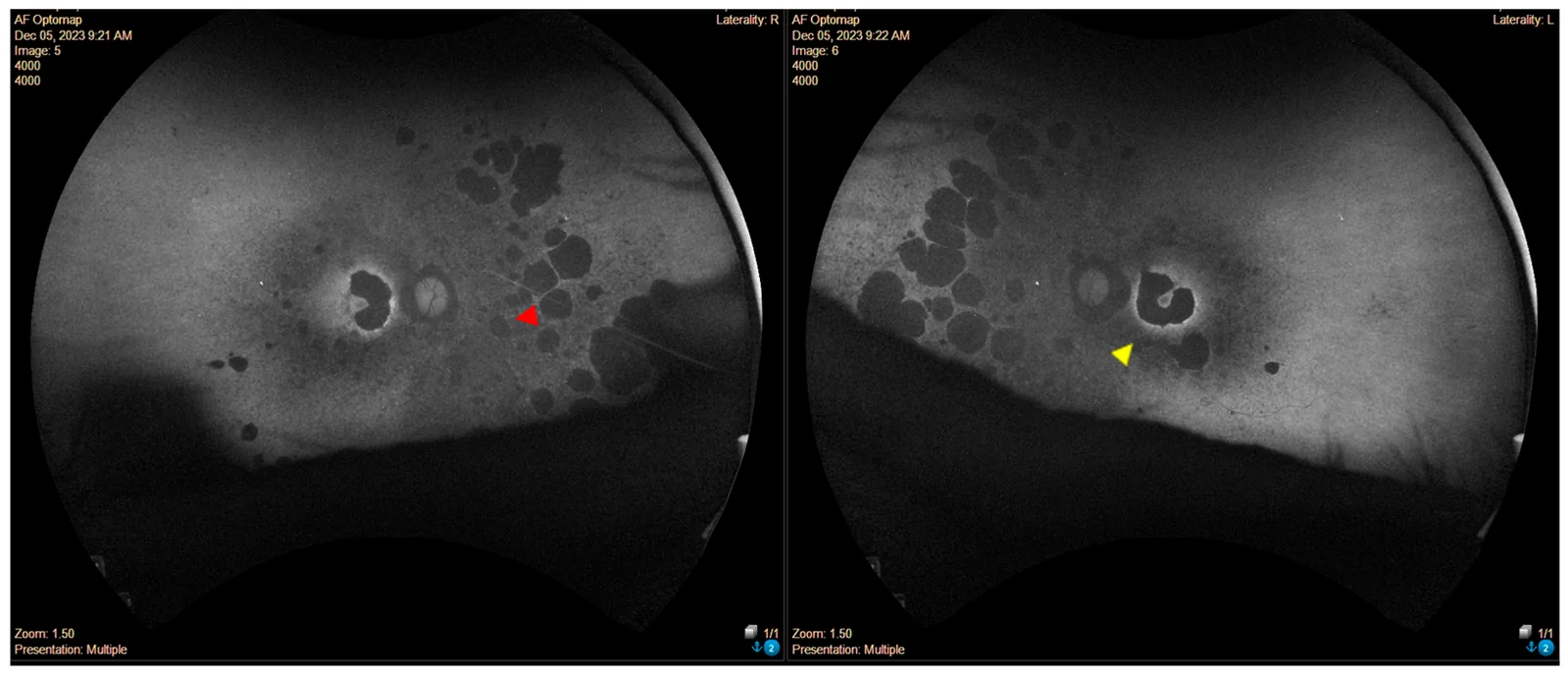
Fundus autofluorescence photos of eyes of patient LUC 15 with *RHO*-associated retinitis pigmentosa. FAF presented macular transition zone and the spatial pattern of RPE dysfunction/atrophy. Parafoveal partial hyperautofluorescent ring (yellow arrowhead), and diffuse freckles of hypo-AF in the midperiphery (red arrowhead).

Seven patients carried pathogenic RHO variants, with c.1040C>T identified in four of the seven patients. All identified pathogenic variants were missense substitutions, consistent with the well-established mutational spectrum of dominant RHO-associated retinopathy. The high recurrence of c.1040 C>T suggests that this allele may represent a recurrent or founder variant within the studied group. Because all of the patients had a pathogenic *RHO* variant compatible with autosomal-dominant inheritance, the molecular diagnostic yield for the *RHO* cohort was 100%. In our cohort of patients with RHO-associated autosomal-dominant RP, FAF demonstrated a macular transition zone and the spatial pattern of RPE dysfunction and atrophy. All of the patients presented a parafoveal hyper-AF ring. FAF showed expanding areas of decreased AF in mottled form, leading to geographic hypo-AF outside the ring that reflected increasing RPE/photoreceptor loss. In one patient, the ring was incomplete and arc-shaped (Figure 11).

### 2.8. EYS Gene

**Figure 12:**
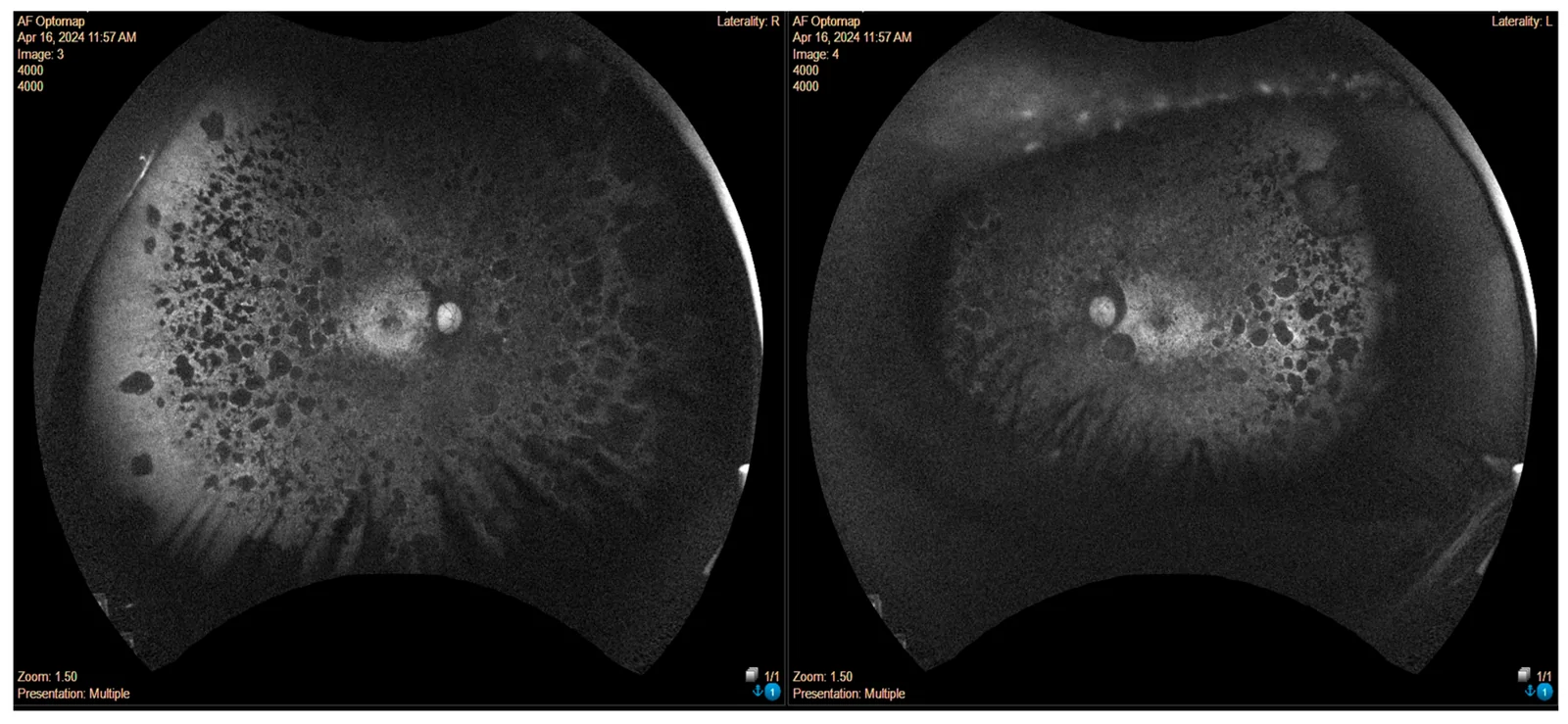
Fundus autofluorescence images of eyes in patient LUC 177 with *EYS* gene-linked retinitis pigmentosa. Hyperautofluorescence complete ring in the macula. Diffuse multifocal, granular hypoautofluorescence extending from the mid- to distal periphery.

Six patients carried *EYS* variants, with a high proportion of truncating and structural variants, including multiple recurrent exon-level deletions. Biallelic pathogenic or likely pathogenic *EYS* variants were identified in 75% of patients, confirming a strong autosomal-recessive disease mechanism. In patients with EYS variants, FAF demonstrated patchy hyperautofluorescent foci within the macula and a complete hyper-AF ring. Bilateral diffuse granular hypo-AF extended from the midperiphery to the distal periphery, with multifocal punctate areas of reduced AF (Figure 12). Bilateral diffuse granular hypo-AF extending from the mid- to distal periphery, with multifocal punctate areas of reduced AF (Figure 12).

### 2.9. PRPF31 Gene

**Figure 13:**
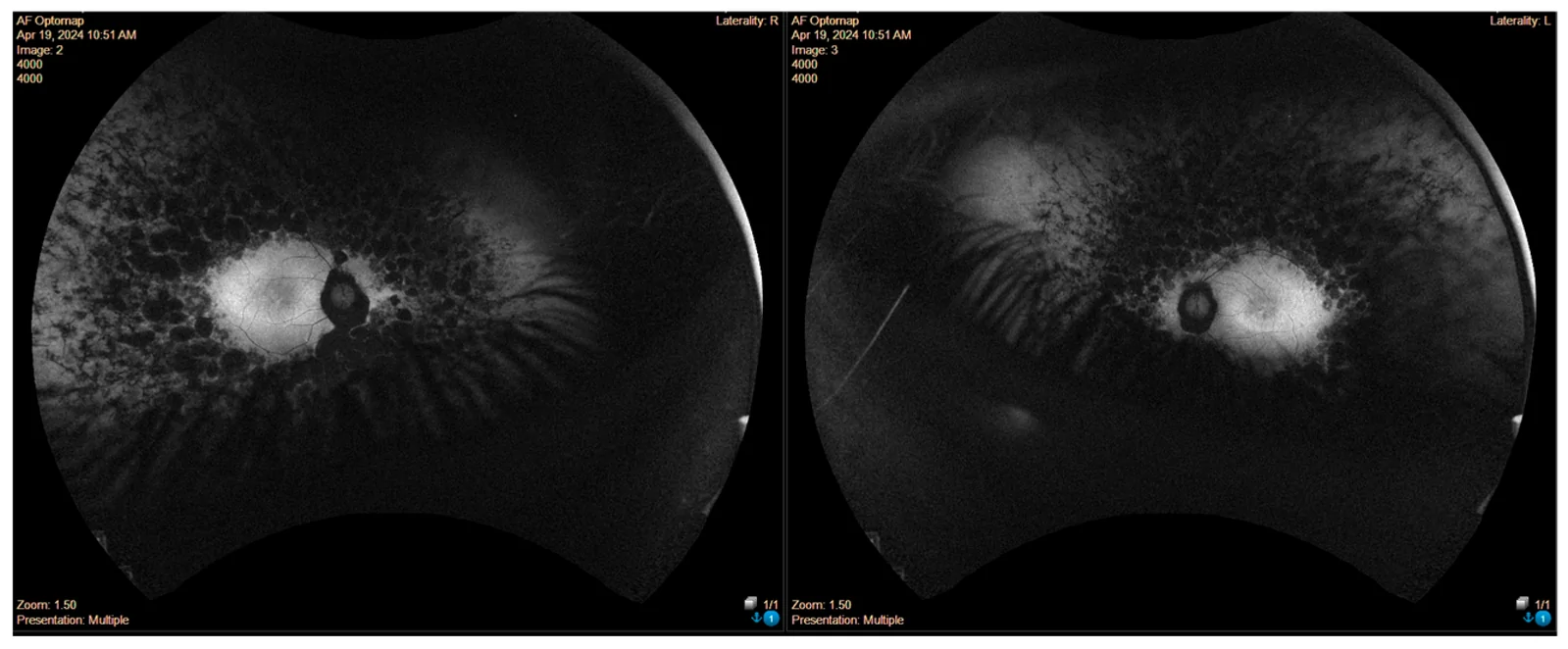
Fundus autofluorescence images of eyes in patient LUC 130 with *PRPF31*-linked retinitis pigmentosa. Well-defined parafoveal hyperautofluorescent complete ring surrounding the fovea. Extensive peripheral hypoautofluorescence with patchy areas of mottled autofluorescence in the periphery.

Alterations in the *PRPF31* gene are the cause of autosomal-dominant RP (adRP), typically through haploinsufficiency (loss-of-function). The cohort included 5 patients with pathogenic PRPF31 variants. The most frequent alteration was exon deletion. Namely, 3 of the 5 patients (60%) had exon 4 deletion, with 1 patient showing exon 8 deletion. FAF demonstrated a well-defined parafoveal hyperautofluorescent complete ring surrounding the fovea. Extensive peripheral hypo-AF corresponding to retinal pigment epithelium atrophy was observed, with patchy areas of mottled AF in the periphery (Figure 13).

### 2.10. CDHR1 Gene

**Figure 14:**
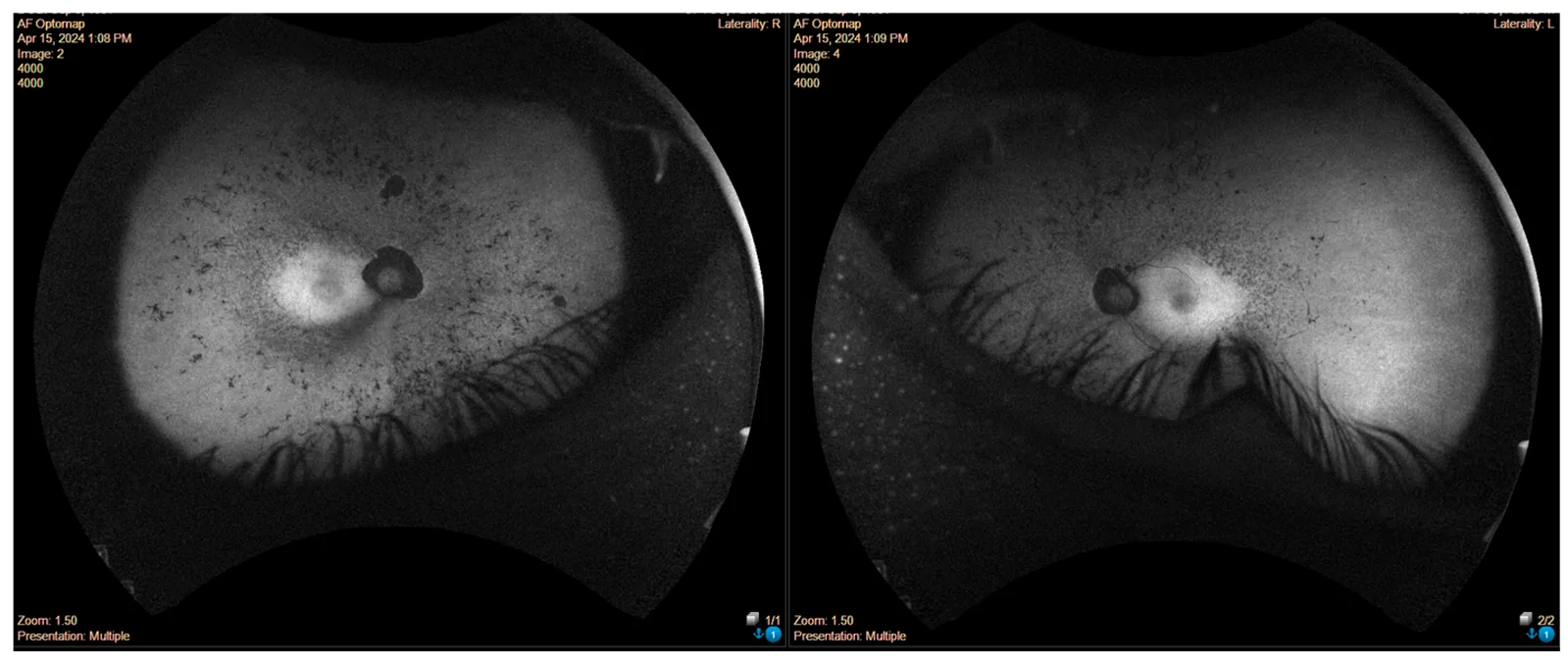
Fundus autofluorescence images of eyes in patient LUC174 with *CDHR1*-linked retinitis pigmentosa. Well-defined hypoautofluorescent macula surrounded by a hyperautofluorescent hyper-AF ring. Patchy peripheral hypoautofluorescence with varying intensity and extent.

The CDHR1 variant c.783G>A was identified in all five patients and was classified as pathogenic or likely pathogenic. One patient additionally carries a pathogenic *ABCA4* variant (c.5882 G>A). Given the established association of *CDHR1* with autosomal-recessive IRDs, the recurrent detection of c.783 G>A suggests that this variant may represent a significant disease-associated allele within the studied cohort. Two patients in this group had the CRD phenotype. FAF showed bilateral symmetrical parafoveal abnormalities in all patients, with a well-defined hypoautofluorescent center corresponding to retinal pigment epithelium atrophy, surrounded by a hyperautofluorescent transition zone (hyper-AF ring). Patchy peripheral hypo-AF of varying intensity was also present (Figure 14).

## 3. Discussion

FAF has become one of the most important examinations for the diagnosis and management of patients with IRDs [15]. FAF is a high-yield adjunct in IRDs, but its main limitations are limited specificity, strong dependence on acquisition/processing, and imperfect structural/functional correlation without multimodal testing. Non-pathognomonic patterns can occur across many IRDs; the same pattern can reflect different genes and different stages of the disease. The data regarding FAF patterns of Polish patients with IRD are scarce. The most relevant is a publication of Wiącek and colleagues [16] describing a cohort of 73 patients with RP. However, this study focused on central FAF patterns, and not all patients were genotyped.

In the present study, we have assessed FAF patterns in ten groups of Polish genotyped patients with IRD phenotypes such as RP, Usher syndrome, and CRD. To date, studies focusing on individual genes have often been limited to the more common genetic causes of RP, including disease associated with RHO or USH2A, because of the limited number of affected patients. The most common FAF pattern observed in our study was a central hyper-AF ring accompanied by peripheral patchy hypo-AF (70%). This hyper-AF ring, which is seen in over half of patients with RP and is undetectable upon fundus examination, has been frequently studied as a marker of disease progression because of its strong correlation with other structural and functional measures [17,18]. Some authors described the ring as either complete or incomplete [16,19]; however, in our patient series, the hyper-AF ring was usually complete. An elevated lipofuscin load in RPE cells has been suggested to be the cause of hyper-AF rings in RP due to enhanced outer-segment degeneration as a precursor to apoptosis [20]. The clinical significance of the width of the hyper-AF ring is unclear. In a study by Trichonas [21], 17 patients with IRDs have been evaluated. Macular hyper-AF was noted in all patients. A ring of hyper-AF was clear in patients with *RHO* and *USH2A* mutations. Moreover, patients with *USH2A* mutations demonstrated a second ring of hyper-AF. In the periphery, patients with *RHO* or *RPGR* variants exhibited hyper-AF with patchy areas of hypo-AF. Patients with *USH2A* variants had a distinctive pattern of diffuse and homogeneous peripheral hypo-AF. Our results are consistent with these observations. Focal RPE atrophy is likely represented by small hypo-AF lesions.

Double hyper-AF rings have been described in diseases associated with variants in *RHO*, *RPGR*, *USH2A*, as well as *NR2E3*, while irregular rings are more common with variants in *RHO*, *RP1*, and *EYS* [22,23,24]. The double concentric hyper-AF ring was also previously described as a possible pathognomonic marker for *NR2E3*-linked RP. It was not, however, confirmed by Dowd-Schoeman and co-workers [24], who distinguished similar FAF appearances in different IRDs, even in small patient groups. The double ring formation described by Escher et al. [25] correlates with the FAF images taken in our cohort, with smaller rings found in the perifoveal area while the larger ones were found alongside vascular arcades. Among patients in the *NR3E3* group, their FAF images had the most uniform appearance in the whole cohort. Fakin and colleagues [26] described FAF patterns in *USH2A*-related RP, showing that the double hyper-AF ring was found only in 3 patients compared to the 24 single hyper-AF rings found. In our cohort of the *USH2A* group, only one patient was found having a double hyper-AF ring.

Mottled to confluent hypo-AF in the posterior pole was the second most prevalent FAF pattern in our cohort, presented in 19 of 22 *ABCA4* patients. Macular involvement was present in 3 *ABCA4* patients, featuring central hypo-AF with a surrounding hyper-AF border (hyper-AF ring) or an atrophy-with-junctional-zone appearance. Peripapillary sparing, the relative preservation of the AF signal around the optic disk, was present in 2 patients. ABCA4-related retinopathy has previously been described as manifesting in three phenotypic groups: macular involvement, peripapillary sparing, and flecks [27]. Progressive deterioration of cellular layers originating in the central macula is a canonical feature of *ABCA4*-associated retinopathy. Flecks are extracellular accumulations of unphagocytized outer segments. The etiology of peripapillary sparing is unknown, although several theories have been proposed, including the disk membrane-load hypothesis, light-load hypothesis, lipofuscin-clearance hypothesis, and neurotrophic factor hypothesis [27].

In patients with PROM1 variants, a common feature of all AF images was a macular hypo-AF with a subtle hyper-AF border surrounding the area of atrophy, as also observed in ABCA4-related retinopathy. The scans differ primarily in the severity of fluorescence defects in the midperiphery and far periphery of the retina. These findings were consistent with published descriptions of FAF in PROM1-associated retinal disease [28].

Seven patients carried pathogenic variants in *RHO*, with c.1040 C>T accounting for more than half of all *RHO*-positive cases (4 of 7 cases). All pathogenic variants identified were missense substitutions, consistent with the well-established mutational spectrum of dominant *RHO*-associated retinopathy. The high recurrence of c.1040 C>T suggests that this allele may represent a recurrent or founder variant within the studied group. Because all of the patients had a pathogenic *RHO* variant compatible with autosomal-dominant inheritance, the molecular diagnostic yield for the *RHO* cohort was 100%. FAF images in patients with RHO variants usually show a parafoveal or macular hyper-AF ring or arc. The formation of a hyper-AF arc rather than a complete ring is mainly caused by an asymmetric cone distribution [29]. In our RHO group, only one patient presented arch-shaped changes in AF.

FAF in *RPGR*-associated X-linked retinopathy most commonly shows a macular hyper-AF ring (or annulus) that represents a transition zone between relatively preserved central photoreceptors and more peripheral degeneration. Outside the hyper-AF ring, FAF often becomes increasingly abnormal with mottled to patchy/confluent hypo-AF consistent with RPE/photoreceptor loss. In patients with the cone–rod phenotype, we observed central confluent or patchy macular atrophy (hypo-AF) surrounded by hyper-AF. This is consistent with findings described by Padhy et al. [30].

Five patients carried RP1 variants. In our patients with RP1-associated RP, FAF revealed hyper-AF perimacular complete rings and macular atrophy. The peripheral hypo-AF pattern, ranging from mottled to confluent geographic hypo-AF, was consistent with findings reported in the literature [31].

On FAF, *EYS*-RP has been described as showing diffuse low AF in the peripheral retina with a ring of AF enhancement at the macula hyper-AF ring. In 2025, Kominami et al. described an additional FAF pattern, termed the “infinity sign,” in Japanese patients with EYS-associated RP [32]. They concluded in their paper that the infinity sign has clinical utility in identifying patients with EYS-associated disease. In our cohort with six confirmed cases of an *EYS* gene variant, none presented an infinity FAF pattern.

The group of *CDHR1* retinal dystrophy was completely associated with the c.783 G>A variant. The FAF images showed a typical hyper-AF ring. Our results are consistent with Charbel’s finding [33], who reported a FAF pattern in the *CDHR1* c783 G>A-related RP. On the other hand, Ba-Abbad R et al. [34] reported that FAF imaging revealed evidence of mild structural abnormalities among the examined patients. They emphasize the role of additional examinations: OCT and electroretinography, with the findings indicated severe generalized cone–rod dysfunction.

There is stage dependence of the FAF image “ceiling” or “floor” effect. In advanced disease, extensive confluent RPE loss may result in uniform hypo-AF areas, producing a “floor effect” that limits the ability to track changes beyond changes in lesion size. Conversely, in early disease stages, abnormalities may be detected by ERG, dark adaptometry, or OCT despite minimal FAF changes. Furthermore, a “normal FAF” does not exclude clinically meaningful IRD. However, FAF is an established primary outcome measure in clinical trials for IRDs, as it enables the assessment of hyper-AF ring size or area of hypo-AF atrophy.

Blockage or true RPE loss can be misdiagnosed as hemorrhage, exudate, pigment clumping, fibrosis, and thickened retina that can cause hypo-AF without primary atrophy—these often require OCT correlation. On the other hand, physiologic foveal hypo-AF can mask central disease or complicate the interpretation of “foveal sparing”. Moreover, lid and lash artifacts and peripheral projection distortion can mimic peripheral hypo-AF. Therefore, repeatable image acquisition, including consistent steered fields and centering, is important when monitoring IRDs over time. As reported in the literature, phenotypic variability limits the interpretation of the genotype–phenotype associations described in our study. This can occur with the stage of disease for progressive conditions, different variants in the same gene, or different modes of inheritance. The phenotype variability in RPGR, for example, can be associated with RP, Leber congenital amaurosis, macular dystrophy, and CRD [35]. FAF provides a topography of the AF signal but does not directly measure photoreceptor function or the photoreceptor microstructure (OCT ellipsoid zone). Quantitative FAF (qAF) can be informative in selected disorders but requires strict standardization (calibration, uniform acquisition, comparable media clarity) [36,37]. Our study was limited by the lack of quantitative analysis, focusing only on FAF pattern descriptions. Another limitation was a small sample size for certain genes and a lack of longitudinal data.

Several biomarkers for potential clinical use, such as changes in hyper-AF ring size or area of hypo-AF atrophy, are increasingly being considered as potential outcome measurements in clinical trials. As molecular diagnosis improves and our understanding of IRDs deepens, FAF will likely remain a mainstay of clinical practice and further research.

## 4. Materials and Methods

### 4.1. Patients

This is a retrospective study of patients with IRDs selected for Lublin Digital Union project performed at the Department of the General and Pediatric Ophthalmology of the Medical University of Lublin, Poland. The study was formally approved by the local Institutional Ethics Committee of the Medical University of Lublin (nr KE-0254/260/12/2022). Written informed consent was obtained from patients prior to data collection. This research was approved and conducted in accordance with the principles outlined in the Declaration of Helsinki.

### 4.2. Clinical Assessment

Inclusion criteria were the clinical diagnosis of retinitis pigmentosa (RP), Usher syndrome or cone–rod dystrophy. All patients were examined in the out-patient clinic and underwent a detailed family and medical history interview, fundoscopy, slit lamp examination and visual acuity testing. Moreover, patients underwent functional examinations, such as visual field examination, as well as electroretinography to confirm the diagnosis of IRD.

All patients underwent wide-field imaging (Optos plc, Dunfermline, Scotland, UK) including wide-field fundus photograph and FAF after pupil-widening.

The FAF images were downloaded from the device in a pseudo-anonymized manner and assigned a study identification number. Some of the authors (A.P.-B. and K.N.) examined the FAF images and described the characteristics of the pattern in each image. Masked grading was applied, so both graders were blinded to the patient’s clinical details, genetic diagnoses, and the other observer’s assessment. If the two observers disagreed on a qualitative pattern, a consensus review was triggered. A third senior expert grader resolved the disagreement.

Images were categorized by FAF patterns, such as hyper-AF (increased signal indicating lipofuscin accumulation or stress) and hypo-AF (decreased signal indicating RPE atrophy or loss).

### 4.3. Genotyping Analysis

Genetic testing was performed in CeGaT laboratory in Tübingen, Germany. Genomic DNA was extracted from the peripheral blood of each participant using the commercial QIA Symphony DSP DNA Mini Kit (Qiagen N.V., Venlo, The Netherlands) according to the manufacturer’s protocol and subsequently quantified on a Gemini XPS Dual-Scanning Microplate Spectrofluorometer (Molecular Devices Corporation, Sunnyvale, CA, USA). The extracted DNA was used for whole-exome sequencing (WES) on the Illumina NovaSeq6000 platform (Illumina, Inc., San Diego, CA, USA) following target enrichment via solution-based hybridization using a custom WES panel (CeGaT Exome Xtra^®^ v.5) developed by Twist Bioscience (South San Francisco, CA, USA). Additionally, known non-coding pathogenic variants (UTR, intronic, and intergenic) were enriched and assessed according to ClinVar, HGMD, and CeGaT’s internal database. Sequencing reads were processed using bcl2fastq2, aligned to the reference genome hg19 (Skewer, BWA), and analyzed for SNVs, small indels and copy number variations (CNVs). CNVs were calculated based on uniquely mapping, non-duplicated, high-quality reads using an internally developed method based on sequencing coverage depth. Variants were additionally analyzed using in silico panels for RP, consistent with the patients’ phenotypes Variant interpretation was based on the clinical information available at the time of analysis, and variants were classified according to ACMG/ACGS 2020 guidelines (version 4.01).

Genetic testing established a conclusive molecular diagnosis in 132 of 203 cases (65.%). In 37 patients, molecular analysis did not determine the genetic basis of the retinal disease, which represented 18.23% of outcomes in our cohort. An inconclusive outcome was determined in 34 (16.75%) patients. In these cases, the analysis identified a variant of uncertain significance (VUS), meaning there is insufficient information to determine its clinical significance.

**Figure 15:**
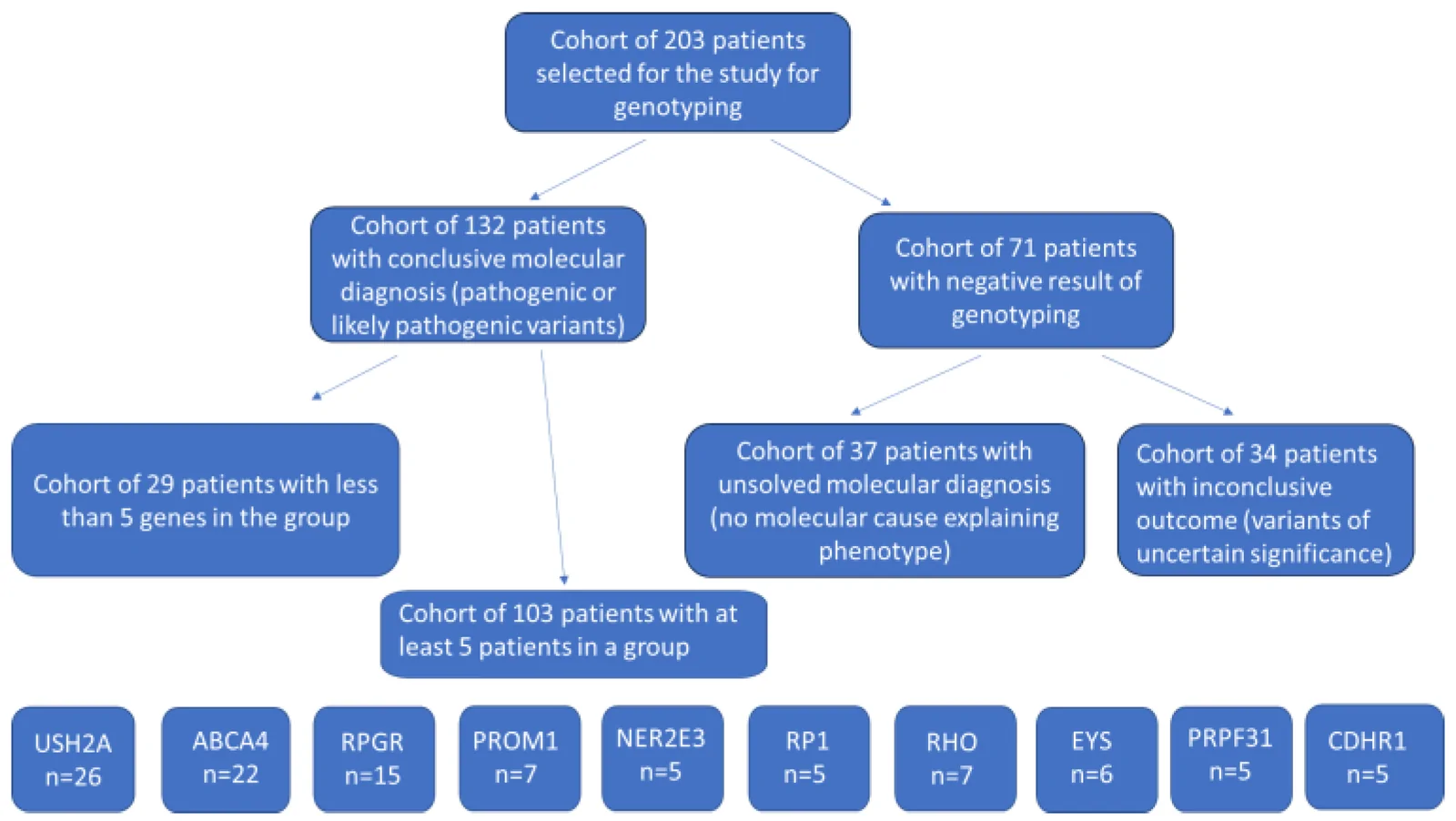
Diagram showing the selection algorithm of patients for this study.

The 132 patients included in this study had pathogenic variants spanning different genes associated with IRDs, all having either autosomal-dominant, autosomal-recessive or X-linked patterns of inheritance (see Supplementary Material). The FAF images were then compiled into groups based on genotype. If a cohort included five or more representative cases of a particular gene, the patients were included in this study. The basis for this criterium was the fact that IRDs are genetically heterogeneous and clinically variable. A minimum of five patients helps to establish whether a specific FAF pattern is truly characteristic of the genotype or merely an isolated occurrence. Studies focusing on individual genes are often limited to the more common genetic causes of RP, such as RHO or USH2A, because of the small number of affected patients [7]. Ultimately, ten groups within 103 patients were established: *USH2A*, * ABCA4*, *RPGR*, *PROM1*, *NR2E3*, *RP1*, *RHO*, *EYS*, *PRPF31* and *CDHR1* (Figure 15). The mean age of included patients was 42 years, and there were 45 males included in the final cohort. There was an additional group of 3 patients with the PDE6B gene, and 8 pairs of patients with the following genes: *AGBL5*, *BBS5*, *IMPG2*, *MERKT*, *NRL*, *PRPF3*, *PRPH2*, *ALPK1.* The remaining genes identified in association with RP were detected in a single patient each. Detailed information regarding the entire cohort is included in a paper published recently by our team [38].

In this study, a “resolved molecular diagnosis” was defined exclusively as cases in which pathogenic or likely pathogenic (P/LP) variants, classified according to ACMG/ACGS criteria, were deemed sufficient to explain the phenotype and consistent with the expected pattern of inheritance: two variants in the same gene for autosomal-recessive (AR) disorders, one variant for autosomal-dominant (AD) disorders, and one hemizygous variant for X-linked disorders. Patients with a single heterozygous variant in a gene associated with AR inheritance were therefore not considered “molecularly solved” solely on this basis. These criteria have been described in a previous publication [38].

No family cosegregation analysis has been performed. Consequently, it was not possible to formally confirm whether the two heterozygous variants detected in patients with AR diseases were in an in-trans configuration, which would confirm the presence of variants on two different alleles, with one allele typically inherited from each parent. Molecular diagnoses were therefore based on the established ACMG/ACGS criteria and the available clinical and molecular data. The genotype–phenotype relationships obtained require further verification, preferably through family studies that allow for the confirmation of segregation and the allelic pattern of the variants.

## Data Availability

The original contributions presented in this study are included in the article/Supplementary Materials. Further inquiries can be directed to the corresponding authors.

## Supplementary Materials

The following supporting information can be downloaded at: https://www.mdpi.com/article/10.3390/ijms27219744/s1.
