# Supplementary material for: Wide-Field Fundus Autofluorescence Patterns in a Polish Cohort of Genotyped Patients with Phenotype of Retinitis Pigmentosa, Usher Syndrome and Cone–Rod Dystrophy
Source: Int J Mol Sci. 2026 Oct 30;27(21):9744. doi: 10.3390/ijms27219744 (PMC13763356; doi:10.3390/ijms27219744)
Supplement: Supplementary file 1 [file ijms-27-09744-s001.zip › ijms-4522229-supplementary final.pdf]

Supplementary Materials

Table containig information in regard to gender, phenotype of examined cohort of patients. Fundus autofluorescence image and classification, gene and pathogenic variants and classification of the variant are also included. Only molecularly solved variants have been included. Novel variants are bolded. Abbreviations: AF-autofluorescence, F-female, M-male, het-heterozygous, homo-homozygous, AR-autosomal recessive, AD-autosomal dominant, XL-X-linked, XLR-X-linked recessive, USH-Usher syndrome, RP-retinitis pigmentosa, CRD-cone-rod dystrophy.

Patient study number	Gender	Age	Pheno type	Fundus autofluorescence image	Fundus autofluorescence classification	Gene and reference trancript	HGVS type	Classification	Protein-level notation	Zygozity	Inheritance pattern
LUC23	F	67	USH	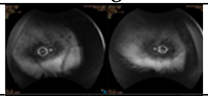	Hyper AF ring	USH2A NM_206933.4	c.11864G>A	Pathogenic	p.Trp3955*	het	AR
							c.2231G>A	Pathogenic /Likely Pathogenic	p.Cys744Tyr	het	AR
LUC24	M	23	USH	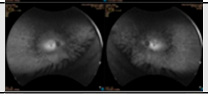	Focal hypo AF and hyper AF ring	USH2A NM_206933.4	c.11864G>A	Pathogenic	p.Trp3955*	hom	AR
LUC25	F	27	RP	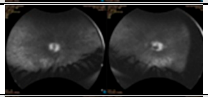	Focal hypo AF and hyper AF ring	USH2A NM_206933.4	c.12574C>T	Pathogenic	p.Arg4192Cys	het	AR
							c.2610C>A	Pathogenic	p.Cys870*	het	AR
LUC29	F	57	RP	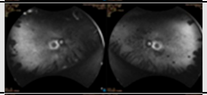	Focal hypo AF and hyper AF ring	USH2A NM_206933.4	c.11864G>A;	Pathogenic	p.Trp3955*	het	AR
LUC53	M	53	USH	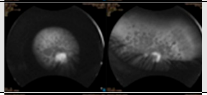	Focal hypo AF and hyper AF ring	USH2A NM_206933.4	c.8682-9A>G	Pathogenic	Nd	homo	AR
LUC56	F	69	RP	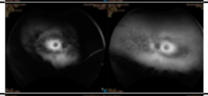	Focal hypo AF and hyper AF ring	USH2A NM_206933.4	c.10073G>A	Pathogenic	p.Cys3358Tyr	het	AR
							c.176G>A	Pathogenic /Likely Pathogenic	p.Gly59Glu	het	AR
LUC58	F	27	RP	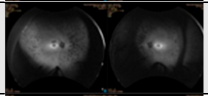	Focal hypo AF and hyper AF ring	USH2A NM_206933.4	c.638A>G	VOUS	p.His213Arg	hom	AR
LUC63	F	50	RP	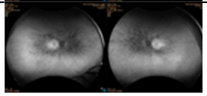	Focal hypo AF and hyper AF ring	USH2A NM_206933.4	c.11864G>A	Pathogenic	p.Trp3955*	het	AR
							c.12268C>A	Pathogenic /Likely Pathogenic	p.Pro4090Tyr	het	AR
							c.44A>G	VOUS/Likely Benign	p.Gln15Arg	het	AR
LUC65	F	42	RP	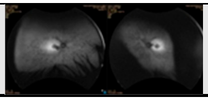	Hyper AF ring	USH2A NM_206933.4	**deletion of exons 21-23 novel variant**	Pathogenic/Likely Pathogenic	nd	het	AR

LUC70	M	45	RP	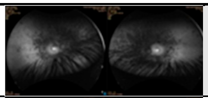	Focal hypo AF and hyper AF ring	USH2A NM_206933.4	c.13010C>T	Pathogenic	p.Thr4337Met	het	AR
LUC71	F	56	RP	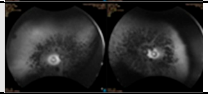	Focal hypo AF and hyper AF ring	USH2A NM_206933.4	c.11864G>A	Pathogenic	p.Trp3955*	het	AR
							c.2276G>T	Pathogenic	p.Cys759Phe	het	AR
LUC73	F	19	USH	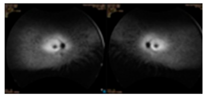	Focal hypo AF and hyper AF ring	USH2A NM_206933.4	**c.9067_9070dup novel variant**	Pathogenic/Likely Pathogenic	p.Leu3024Hisfs*30	het	AR
							**deletion of exons 21-23 novel variant**	Pathogenic	c.4627+25435_4987+658del	het	AR
LUC74	M	63	RP	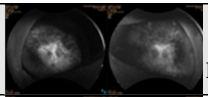	Focal hypo AF and peripapilary sparing	USH2A NM_206933.4	c.2610C>A	Pathogenic	p.Cys870*	het	AR
							c.2915G>A	Pathogenic	p.Cys972Tyr	het	AR
LUC76	F	53	USH	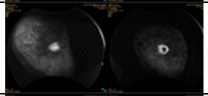	Focal hypo AF and hyper AF ring	USH2A NM_206933.4	c.11864G>A	Pathogenic	p.Trp3955*	het	AR
							c.9424G>T	Pathogenic	p.Gly3142*	het	AR
LUC77	M	33	USH	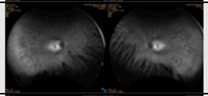	Focal hypo AF and hyper AF ring	USH2A NM_206933.4	c.8682-9A>G	Pathogenic	nd	het	AR
LUC78	F	51	RP	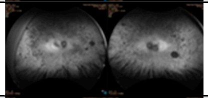	Focal hypo AF and hyper AF ring	USH2A NM_206933.4	c.2610C>A	Pathogenic	p.Cys870*	homo	AR
LUC80	F	44	USH	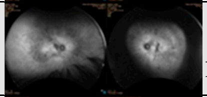	Focal hypo AF and peripapilary sparing	USH2A NM_206933.4	c.11864G>A	Pathogenic	p.Trp3955*	het	AR
LUC120	M	39	RP	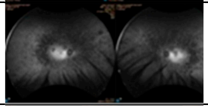	Focal hypo AF and hyper AF ring	USH2A NM_206933.4	**deletion ex 21-23 novel variant**	Likely Pathogenic	nd	hom	AR
LUC153	M	44	RP	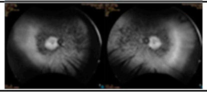	Focal hypo AF and double hyper AF ring	USH2A NM_206933.4	deletion of coding exons 21-23	Likely Pathogenic	nd	het	AR
LUC162	F	45	RP	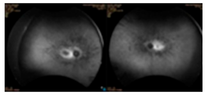	Focal hypo AF and hyper AF ring	USH2A NM_206933.4	c.13335_13347delins CTTG	Pathogenic	p.Glu4445_Ser4449delinsAspLeu	het	AR
							**c.11104T>G novel variant**	VOUS	p.Trp3702Gly	het	AR
LUC163	M	55	RP	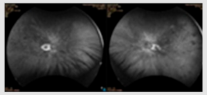	Focal hypo AF and hyper AF ring	USH2A NM_206933.4	c.13335_13347delins CTTG	Pathogenic	p.Glu4445_Ser4449delinsAspLeu	het	AR
							**c.11104T>G novel variant**	VOUS	p.Trp3702Gly	het	AR

LUC192	F	39	RP	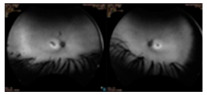	hyper AF ring	USH2A NM_206933.4	c.12575G>A	Pathogenic/Likely Pathogenic	p.Arg4192His	het	AR
							deletion of exons 21-23	Pathogenic/Likely Pathogenic	nd	het	AR
LUC193	F	33	RP	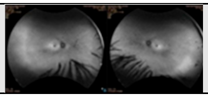	hyper AF ring	USH2A NM_206933.4	c.920_923dup	Pathogenic	p.His308Glnfs*16	het	AR
							c.10732A>C	Likely Pathogenic/VOUS	p.Ser3578Arg	het	AR
LUC200	F	46	RP	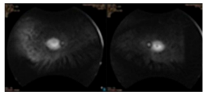	Focal hypo AF and hyper AF ring	USH2A NM_206933.4	**c.4368_4369delTT novel variant**	Likely Pathogenic	p.Ser1457Glyfs*19	het	AR
							c.13335_13347delins CTTG	Pathogenic	p.Glu4445_Ser4449delinsAspLeu	het	AR
LUC203	M	66	RP	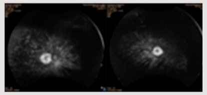	Focal hypo AF and hyper AF ring	USH2A NM_206933.4	deletion of coding exons 9-10	Pathogenic/Likely Pathogenic	nd	het	AR
							c.13339A>G	Pathogenic/Likely Pathogenic	p.Met4447Val	het	AR
LUC110	M	47	RP	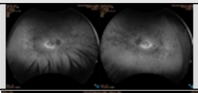	Focal hypo AF and hyper AF ring	USH2A NM_000350.3	deletion exons 21-23	Pathogenic/Likely Pathogenic	nd	het	AR
LUC8	F	59	RP	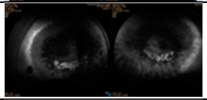	Focal hypo AF	ABCA4 NM_000350.3	c.5196+1G>A	Pathogenic	nd	het	AR
							c.454C>T	Pathogenic	p.Arg152*	het	AR
LUC9	M	50	RP	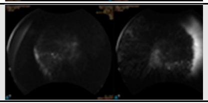	Focal hypo AF	ABCA4 NM_000350.3	c.5196+1G>A	Pathogenic	nd	het	AR
							c.454C>T	Pathogenic	p.Arg152*	het	AR
LUC18	M	13	RP	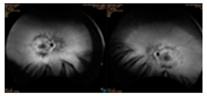	flecks	ABCA4 NM_000350.3	c.3113C>T	Pathogenic/Likely Pathogenic	p.Ala1038Val	het	AR
							c.1622T>C	Pathogenic	p.Leu541Pro	het	AR
							c.768G>T	Pathogenic	p.Val256=	het	AR
LUC34	F	48	RP	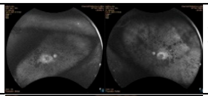	Focal hypo AF with hyper AF ring	ABCA4 NM_000350.3	c.4253+43G>A	Pathogenic/Likely Pathogenic	no	het	AR
LUC39	F	65	RP	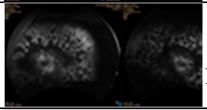	Focal hypo AF and peripapilary sparing	ABCA4 NM_000350.3	c.4919G>A	Pathogenic	p.Arg1640Gln	het	AR
							c.768G>T	Pathogenic	p.Val256=	het	AR
LUC43	F	35	RP	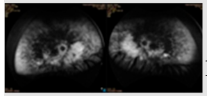	Focal hypo AF and peripapilary sparing	ABCA4 NM_000350.3	c.3113C>T	Pathogenic/Likely Pathogenic	p.Ala1038Val	het	AR
							c.1622T>C	Pathogenic	p.Leu541Pro	het	AR
							c.1022A>G	Pathogenic/Likely Pathogenic	p.Glu341Gly	het	AR

LUC64	F	37	RP	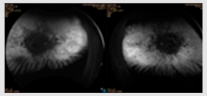	Focal hypo AF	ABCA4 NM_000350.3	c.1622T>C;3113C>T	pathogenic	p.Leu541Pro	het	AR
							c.3113C>T	Pathogenic/Likely Pathogenic	p.Ala1038Val	het	AR
							c.4469G>A	pathogenic	p.Cys1490Tyr	het	AR
LUC68	F	11	CRD	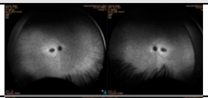	Hypo AF and hyper AF ring	ABCA4 NM_000350.3	c.1496G>A	Pathogenic	p.Trp499*	het	AR
							c.454C>T	Pathogenic	p.Arg152*	het	AR
LUC86	F	11	CRD	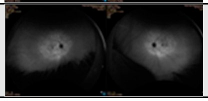	flecks	ABCA4 NM_000350.3	c.5929G>A	Pathogenic	p.Gly1977Ser	het	AR
							c.5714+5G>A	Pathogenic	no	het	AR
LUC89	M	48	RP	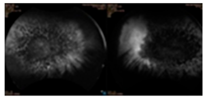	Focal hypo AF	ABCA4 NM_000350.3	c.3113C>T	Pathogenic/Likely Pathogenic	p.Ala1038Val	het	AR
							c.1622T>C	Pathogenic	p.Leu541Pro	het	AR
							**c.3806T>C novel variant**	Pathogenic/Likely Pathogenic	p.Leu1269Pro	het	AR
LUC92	M	42	CRD	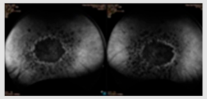	Focal hypo AF	ABCA4 NM_000350.3	c.5512C>T	Pathogenic	p.His1838Tyr	het	AR
							c.3113C>T	Pathogenic/Likely Pathogenic	p.Ala1038Val	het	AR
							c.1622T>C	Pathogenic	p.Leu541Pro	het	AR
LUC101	M	47	RP	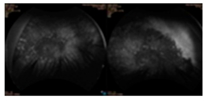	Focal hypo AF	ABCA4 NM_000350.3	c.3259G>A	Pathogenic	p.Glu1087Lys	het	AR
							c.3113C>T	Pathogenic/Likely Pathogenic	p.Ala1038Val	het	AR
							c.1622T>C	Pathogenic	p.Leu541Pro	het	AR
LUC108	M	34	RP	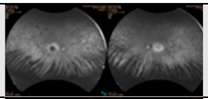	Hyper AF ring and focal hypo AF	ABCA4 NM_000350.3	c.5882G>A	Pathogenic	p.Gly1961Glu	het	AR
LUC116	M	33	RP	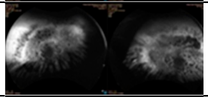	Focal hypo AF	ABCA4 NM_000350.3 NM_000350.3	c.2626C>T	Pathogenic	p.Gln876*	het	AR
							c.1937+1G>A	Pathogenic	no	het	AR
LUC117	M	33	RP	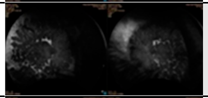	Focal hypo AF	ABCA4 NM_000350.3	c.2626C>T	Pathogenic	p.Gln876*	het	AR
							c.1937+1G>A	Pathogenic	no	het	AR
LUC123	F	67	RP	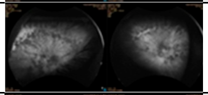	Focal hypo AF	ABCA4 NM_000350.3	c.2564G>A	Pathogenic	p.Trp855*	het	AR
							c.61C>T	Pathogenic	p.Gln21*	het	AR
LUC124	F	48	RP	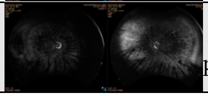	Focal hypo AF with peripapillary sparing	ABCA4 NM_000350.3	c.1622T>C	Pathogenic	p.Leu541Pro	hom	AR
							c.3113C>T	Pathogenic	p.Ala1038Val	hom	AR
LUC134	F	27	RP	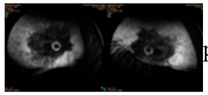	Focal hypo AF with peripapillary sparing	ABCA4 NM_000350.3	c.319C>T	Pathogenic	p.Arg107*	het	AR
							c.1622T>C	Pathogenic	p.Leu541Pro	het	AR
							c.3113C>T	Pathogenic	p.Ala1038Val	het	AR

LUC140	M	41	RP	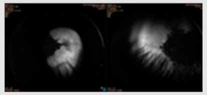	Focal hypo AF	ABCA4 NM_000350.3	c.710T>C<br>c.1622T>C<br>c.3113C>T	Likely Pathogenic<br>Pathogenic<br>Pathogenic/Likely Pathogenic	p.Leu237Pro<br>p.Leu541Pro<br>p.Ala1038Val	het<br>het<br>het	AR<br>AR<br>AR
LUC142	M	48	RP	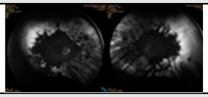	Focal hypo AF	ABCA4 NM_000350.3	c.1988G>A	Pathogenic	p.Trp663*	het	AR
LUC143	F	48	RP	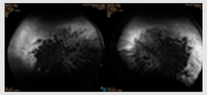	Focal hypo AF	ABCA4 NM_000350.3	c.4919G>A<br>c.1622T>C<br>c.3113C>T	Pathogenic<br>Pathogenic<br>Pathogenic/Likely Pathogenic	p.Arg1640Gln<br>p.Leu541Pro<br>p.Ala1038Val	het<br>het<br>het	AR<br>AR<br>AR
LUC179	F	37	RP	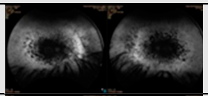	Focal hypo AF	ABCA4 NM_000350.3	c.454C>T	Pathogenic	p.Arg152*	hom	AR
LUC33	M	64	RP	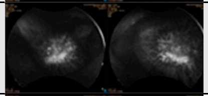	Hyper AF ring and mottled hypo AF	RPGR NM_001034853.2	c.1245+1G>A	Likely Pathogenic	nd	hemi	XLR/XL
LUC46	M	20	RP	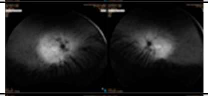	Hyper AF ring and mottled hypo AF	RPGR NM_001034853.2	c.29-2A>C	Likely Pathogenic	nd	hemi	XLR/XL
LUC57	M	69	RP	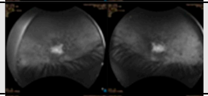	Hyper AF ring and mottled hypo AF	RPGR NM_001034853.2	c.2340_2341delAG	Pathogenic	p.Arg780Serfs*54	hemi	XLR/XL
LUC59	M	40	CRD	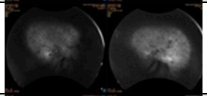	Mottled hypo AF	RPGR NM_001034853.2	**c.1150_1154delTGCTT novel variant**	Pathogenic/Likely Pathogenic	p.Cys384Ilefs*9	hemi	XLR/XL
LUC72	M	46	RP	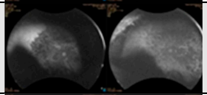	Mottled hypo AF	RPGR NM_001034853.2	c.2405_2406delAG	Pathogenic	p.Glu802Glyfs*32	hemi	XLR/XL
LUC75	M	30	RP	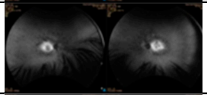	hyper AF ring and mottled hypo AF	RPGR NM_001034853.2	c.2590G>T	Pathogenic/Likely Pathogenic	p.Glu864*	hemi	XLR/XL
LUC79	M	29	RP	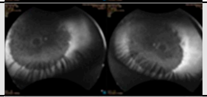	Mottled hypo AF and mottled hypo AF	RPGR NM_001034853.2	c.2442_2445delAGAG	Pathogenic	p.Gly817Lysfs*2	hemi	XLR/XL
LUC93	M	16	RP	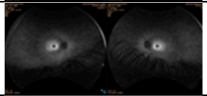	Hyper AF ring and mottled hypo AF	RPGR NM_001034853.2	c.194G>A	Pathogenic	p.Gly65Asp	hemi	XLR/XL
LUC94	M	20	RP	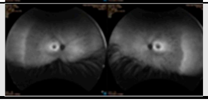	Hyper AF ring and mottled hypo AF	RPGR NM_001034853.2	c.194G>A	Pathogenic	p.Gly65Asp	hemi	XLR/XL

LUC99	M	39	RP	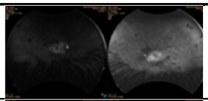	Hyper AF ring and mottled hypo AF	RPGR NM_001034853.2	c.2867delA	Pathogenic	p.Glu956Glyfs*133	hemi	XLR/XL
LUC103	F	36	RP	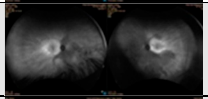	Hyper AF ring and mottled hypo AF	RPGR NM_001034853.2	c.2872delG	Pathogenic/Likely Pathogenic	p.Glu958Lysfs*131	het	XLR/XL
LUC155	M	42	RP	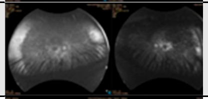	Hyper AF ring and mottled hypo AF	RPGR NM_001034853.2	**c.2597_2598delAA novel variant**	Likely Pathogenic	p.Glu866Glyfs*212	hemi	XL
LUC168	M	65	RP	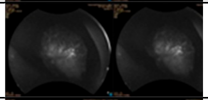	Hyper AF ring and mottled hypo AF	RPGR NM_001034853.2	c.248-10A>G	Likely Pathogenic	nd	hemi	XL
LUC169	M	37	RP	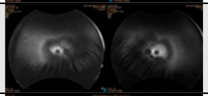	Hyper AF ring and mottled hypo AF	RPGR NM_001034853.2	c.2218G>T	Pathogenic	p.Glu740*	hemi	XL
LUC178	F	29	RP	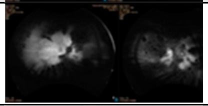	Hyper AF ring with mottled hypo AF	RPGR NM_001034853.2	c.2966delA	Pathogenic	p.Glu989Glyfs*100	het	XL
LUC67	M	29	RP	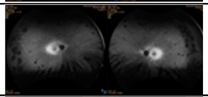	Hyper AF ring and mottled hypo AF	PROM1 NM_006017.3	c.1902C>G	Pathogenic	p.Tyr634*	het	AD, AR
							c.1767G> A	Pathogenic/Likely Pathogenic	p.Glu589=	het	AD, AR
LUC98	F	45	RP	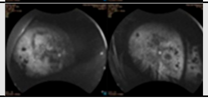	Mottled hypo AF	PROM1 NM_006017.3	c.1767G>A	Pathogenic/Likely Pathogenic	p.Glu589=	hom	AD, AR
LUC112	M	39	RP	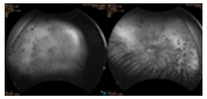	Mottled hypo AF	PROM1 NM_006017.3	c.1301+2T>C	Pathogenic/Likely Pathogenic	nd	het	AD, AR
							c.1454+4_1454+7delAGT	VOUS	nd	het	AD, AR
LUC113	F	33	RP	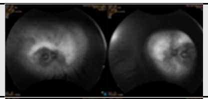	Mottled hypo AF	PROM1 NM_006017.3	c.1301+2T>C	Pathogenic/Likely Pathogenic	nd	het	AD, AR
LUC114	F	44	RP	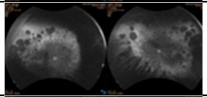	Mottled hypo AF	PROM1 NM_006017.3	c.1301+2T>C	Pathogenic/Likely Pathogenic	nd	hom	AD, AR
LUC126	F	59	RP	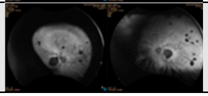	Mottled hypo AF	PROM1 NM_006017.3	c.1902C>G	Pathogenic	p.Tyr634*	het	AD, AR
LUC133	M	38	RP	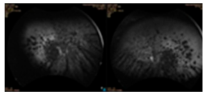	Mottled hypo AF	PROM1 NM_006017.3	c.1902C>G	Pathogenic	p.Tyr634*	hom	AD, AR
							c.604C>T	Pathogenic	p.Arg202*	hom	AD, AR

LUC4	M	48	RP	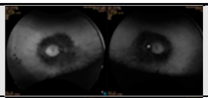	Hyper double AF ring and patchy hypo AF	NR2E3 NM_014249.4	c.227G>A<br>c.481delA	Pathogenic<br>Pathogenic	p.Arg76Gln<br>p.Thr161Hisfs*18	het<br>het	AD, AR<br>AD, AR
LUC146	F	12	RP	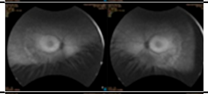	Hyper AF ring and patchy hypo AF	NR2E3 NM_014249.4	c.481delA	Pathogenic	p.Thr161Hisfs*18	homo	AD, AR
LUC147	F	16	RP	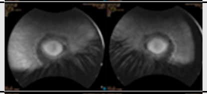	Hyper AF ring and patchy hypo AF	NR2E3 NM_014249.4	c.481delA	Pathogenic	p.Thr161Hisfs*18	homo	AD, AR
LUC166	F	66	USH	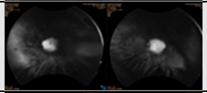	Hyper AF ring and patchy hypo AF	NR2E3 NM_014249.4	c.481delA<br>c.1155dup	Pathogenic<br>Pathogenic	p.Thr161Hisfs*18<br>p.Ile386Hisfs*21	homo<br>het	AD, AR<br>AD, AR
LUC182	M	44	RP	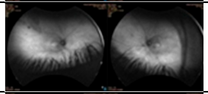	Hyper AF ring and patchy hypo AF	NR2E3 NM_014249.4	c.227G>A	Pathogenic	p.Arg76Gln	homo	AD, AR
LUC6	M	22	RP	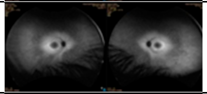	Hyper AF ring and patchy hypo AF	RP1 NM_006269.2	c.2173_2174delinsG	Pathogenic/Likely Pathogenic	p.Ile725Aspfs*13	homo	AD, AR
LUC20	M	40	RP	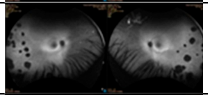	Hyper AF ring and patchy hypo AF	RP1 NM_006269.2	c.3157delT	Pathogenic	p.Tyr1053Thrfs*4	het	AD, AR
LUC38	M	54	RP	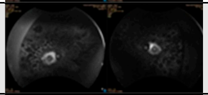	Hyper AF ring and patchy hypo AF	RP1 NM_006269.2	c.2029C>T	Pathogenic	p.Arg677*	het	AD, AR
LUC173	M	46	RP	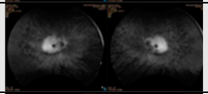	Hyper AF ring and patchy hypo AF	RP1 NM_006269.2	c.2029C>T	Pathogenic	p.Arg677*	het	AD, AR
LUC15	F	74	RP	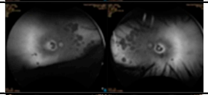	Hyper AF ring and patchy hypo AF	RP1 NM_006269.2	c.2173_2174delinsG	Pathogenic/Likely Pathogenic	p.Ile725Aspfs*13	het	AD, AR
LUC28	F	31	RP	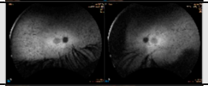	Hyper AF ring and patchy hypo AF	RHO NM_000539.3	c.1040C>T	Pathogenic	p.Pro347Leu	het	AD, AR
LUC51	F	48	RP	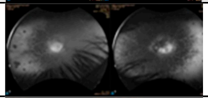	Hyper AF ring and patchy hypo AF	RHO NM_000539.3	c.1040C>T	Pathogenic	p.Pro347Leu	het	AD, AR
LUC52	F	32	RP	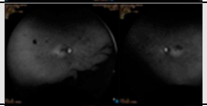	Hyper AF ring and patchy hypo AF	RHO NM_000539.3	c.1040C>T	Pathogenic	p.Pro347Leu	het	AD, AR
LUC60	M	32	CRD	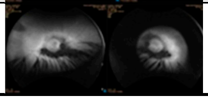	Hyper AF ring and patchy hypo AF	RHO NM_000539.3	c.50C>T	Pathogenic	p.Thr17Met	het	AD, AR

LUC108	M	34	RP	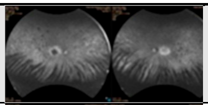	Hyper AF ring and patchy hypo AF	RHO NM_000539.3	c.403C>T	Pathogenic	p.Arg135Trp	het	AD, AR
LUC139	F	45	RP	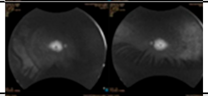	Hyper AF ring and patchy hypo AF	RHO NM_000539.3	c.533A>G	Pathogenic	p.Tyr178Cys	het	AD, AR
LUC177	F	70	RP	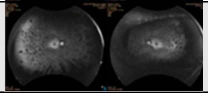	Hyper AF ring and patchy hypo AF	RHO NM_000539.3	c.1040C>T	Pathogenic	p.Pro347Leu	het	AD, AR
LUC198	M	62	RP	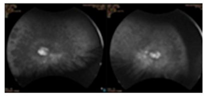	Hyper AF ring and patchy hypo AF	EYS NM_001142800.2	c.96G>A	Pathogenic	p.Trp32*	het	AR
							**deletion of exons 29-32 novel variant**	Pathogenic	nd	het	AR
LUC199	F	23	RP	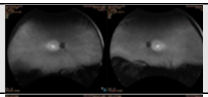	Hyper AF ring and patchy hypo AF	EYS NM_001142800.2	c.1641_1644delTCAG	Pathogenic	p.Ser547Argfs*62	het	AR
							c.9079_9082de	Pathogenic	p.Arg3027Serfs*5	het	AR
LUC176	M	24	RP	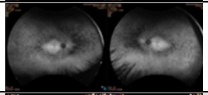	Hyper AF ring and patchy hypo AF	EYS NM_001142800.2	c.6714delT>A	Pathogenic	p.Ile2239Serfs*17	het	AR
LUC137	F	58	RP	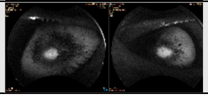	Hyper AF ring and patchy hypo AF	EYS NM_001142800.2	c.8206G>C	Likely Pathogenic/VOUS	p.Ala2736Pro	het	AR
LUC100	M	33	CRD	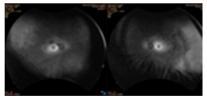	Hyper AF ring and patchy hypo AF	EYS NM_001142800.2	**deletion of exon 29 novel variant**	Likely Pathogenic	nd	het	AR
							c.9079_9082delAGAA	Pathogenic	p.Arg3027Serfs*5	het	AR
LUC30	M	47	RP	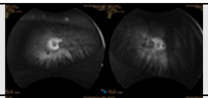	Hyper AF ring and patchy hypo AF	EYS NM_001142800.2	c.7243C>T	Pathogenic	p.Gln2415*	het	AR
							**c.6291_6297dup novel variant**	Pathogenic	p.Pro2100Cysfs*32	het	AR
LUC156	M	36	RP	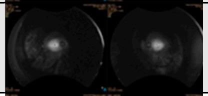	Hyper AF ring and patchy hypo AF	PRPF31	**deletion of exon 8 novel variant**	Likely pathogenic	no	het	AD
LUC130	F	36	RP	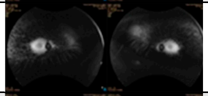	Hyper AF ring and patchy hypo AF	PRPF31	**deletion of coding exon 4 novel variant**	Likely pathogenic	no	het	AD
LUC131	M	48	RP	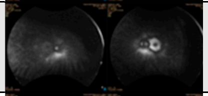	Hyper AF ring and patchy hypo AF	PRPF31	**deletion of coding exon 4 novel variant**	Likely pathogenic	no	het	AD
LUC132	F	28	RP	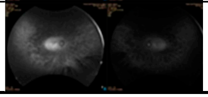	Hyper AF ring and patchy hypo AF	PRPF31	**deletion of coding exon 4 novel variant**	Likely pathogenic	no	het	AD

LUC136	F	33	RP	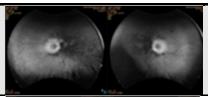	Hyper AF ring and patchy hypo AF	PRPF31	**c.1080_1089del novel variant**	Likely pathogenic	p.Lys361Serfs*5	het	AD
LUC172	F	61	CRD	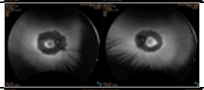	Hyper AF ring and patchy hypo AF	CDHR1 NM_033100.4	c.783G>A	Pathogenic/Likely Pathogenic	p.Pro261=	het	AR
LUC174	F	33	RP	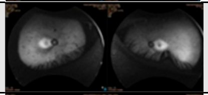	Hyper AF ring and patchy hypo AF	CDHR1 NM_033100.4	c.783G>A	Pathogenic/Likely Pathogenic	p.Pro261=	het	AR
LUC144	M	54	RP	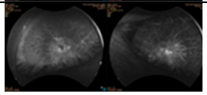	Hyper AF ring and patchy hypo AF	CDHR1 NM_033100.4	c.783G>A	Pathogenic/Likely Pathogenic	p.Pro261=	het	AR
LUC7	F	72	CRD	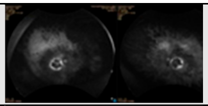	Hyper AF ring and patchy hypo AF	CDHR1 NM_033100.4	c.783G>A	Pathogenic/Likely Pathogenic	p.Pro261=	homo	AR
LUC202	F	39	RP	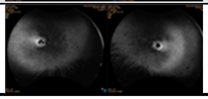	Hyper AF ring and patchy hypo AF	CDHR1 NM_033100.4	c.783G>A	Pathogenic/Likely Pathogenic	p.Pro261=	homo	AR
